# Redox Regulation of Mitochondrial Fission Protein Drp1 by Protein Disulfide Isomerase Limits Endothelial Senescence

**DOI:** 10.1016/j.celrep.2018.05.054

**Published:** 2018-06-19

**Authors:** Young-Mee Kim, Seock-Won Youn, Varadarajan Sudhahar, Archita Das, Reyhaan Chandhri, Henar Cuervo Grajal, Junghun Kweon, Silvia Leanhart, Lianying He, Peter T. Toth, Jan Kitajewski, Jalees Rehman, Yisang Yoon, Jaehyung Cho, Tohru Fukai, Masuko Ushio-Fukai

**Affiliations:** 1 Vascular Biology Center, Medical College of Georgia at Augusta University, Augusta, GA, USA; 2 Department of Medicine (Cardiology), Medical College of Georgia at Augusta University, Augusta, GA, USA; 3 Department of Pharmacology and Toxicology, Medical College of Georgia at Augusta University, Augusta, GA, USA; 4 Department of Physiology, Medical College of Georgia at Augusta University, Augusta, GA, USA; 5 Department of Pharmacology, University of Illinois at Chicago, Chicago, IL, USA; 6 Departments of Medicine (Cardiology) and Pharmacology, University of Illinois at Chicago, Chicago, IL, USA; 7 Department of Physiology and Biophysics, University of Illinois at Chicago, Chicago, IL, USA; 8 Center for Cardiovascular Research, University of Illinois at Chicago, Chicago, IL, USA; 9 Charlie Norwood Veterans Affairs Medical Center, Augusta, GA, USA; 10 Lead Contact

## Abstract

Mitochondrial dynamics are tightly controlled by fusion and fission, and their dysregulation and excess reactive oxygen species (ROS) contribute to endothelial cell (EC) dysfunction. How redox signals regulate coupling between mitochondrial dynamics and endothelial (dys)function remains unknown. Here, we identify protein disulfide isomerase A1 (PDIA1) as a thiol reductase for the mitochondrial fission protein Drp1. A biotin-labeled Cys-OH trapping probe and rescue experiments reveal that PDIA1 depletion in ECs induces sulfenylation of Drp1 at Cys^644^, promoting mitochondrial fragmentation and ROS elevation without inducing ER stress, which drives EC senescence. Mechanistically, PDIA1 associates with Drp1 to reduce its redox status and activity. Defective wound healing and angiogenesis in diabetic or *PDIA1*^+/−^ mice are restored by EC-targeted PDIA1 or the Cys oxidation-defective mutant Drp1. Thus, this study uncovers a molecular link between PDIA1 and Drp1 oxidoreduction, which maintains normal mitochondrial dynamics and limits endothelial senescence with potential translational implications for vascular diseases associated with diabetes or aging.

## In Brief

Kim et al. demonstrate a molecular link between PDIA1 and Drp1 oxidoreduction, which protects against mitochondrial fragmentation and ROS elevation, limiting endothelial senescence. This study provides insights into restoring endothelial PDIA1 function or targeting Drp1 Cys oxidation as potential therapeutic strategies for treating diabetes-associated vascular and metabolic diseases.

## Graphical Abstract

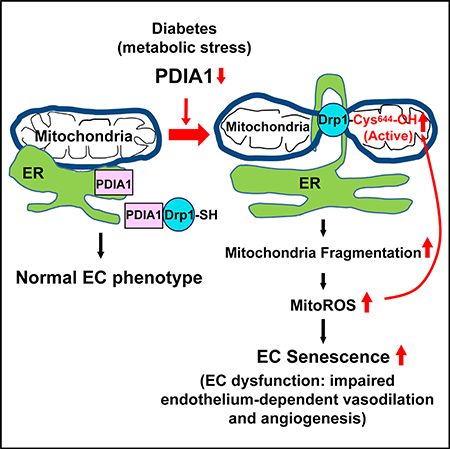

## INTRODUCTION

Endothelial cells (ECs) with senescence impair the integrity of the endothelium in blood vessels, which contributes to vascular aging and cardiovascular and metabolic diseases with unknown mechanisms (Erusalimsky, 2009). In ECs, mitochondria are not the main source of ATP (Vandekeere et al., 2015); they function as reactive oxygen species (ROS) signaling organelles, maintaining EC homeostasis (Kluge et al., 2013). Mitochondrial dynamics are tightly regulated by fusion and fission. Mitofusins (Mfn1 and Mfn2) and optic atrophy 1 (OPA1) mediate fusion of outer and inner membranes, respectively, whereas fission is mediated by the dynamin-related GTPase Drp1 (Chan, 2006). Although mitochondrial fission is involved in physiological function, mitochondrial fragmentation induces excess mitochondrial ROS (mtROS), which results in endothelial dysfunction in pathological conditions such as diabetes (Kluge et al., 2013; Shenouda et al., 2011). Thus, mitochondrial morphology, ROS levels, and endothelial (dys)function are interconnected; however, the mechanism of how redox signals regulate coupling between mitochondrial dynamics and EC function in normal and pathological conditions is poorly understood.

Drp1 is localized in the cytosol in the resting state, and its recruitment to mitochondrial outer membranes induces constriction and scission of mitochondria (Westermann, 2010). Drp1 is also localized at the endoplasmic reticulum (ER)-mitochondria contact site, which plays an important role in the process of mitochondrial fission (Friedman et al., 2011). Drp1 contains a key GTPase activity residue, lysine (K)38, at the N terminus, and the GTP hydrolysis-defective mutant (K38A) acts as a dominant-negative (DN) (Elgass et al., 2013; Westermann, 2010). Post-translational modifications (PTMs) of Drp1, such as phosphorylation at Ser^616^ or Ser^637^, are important mechanisms for regulating mitochondrial fission (Elgass et al., 2013). In addition, nitric oxide (NO) has been shown to induce S-nitrosylation of Drp1, which increases Drp1 GTPase activity and mitochondrial fragmentation in neurodegenerative diseases (Cho et al., 2009). However, this evidence seems to be controversial (Bossy et al., 2010). Another redox-sensitive PTM is ROS-mediated modification of reactive cysteine thiol (SH) to form cysteine sulfenic acid (Cys-OH), termed protein sulfenylation, which is a key initial readout of redox signaling (Paulsen and Carroll, 2010; Poole and Nelson, 2008). However, a role of Drp1 Cys oxidation in mitochondrial dynamics and endothelial function has never been reported.

Protein disulfide isomerases (PDIs) have at least 21 families in eukaryotes and function as thiol oxidoreductases that catalyze thiol oxidation, reduction, or isomerization during protein folding in the ER (Benham, 2012; Xu et al., 2014). A reduced form of PDI functions as a reductase, whereas an oxidized PDI acts as an oxidase to promote disulfide bond formation with specific substrates to regulate their catalytic activity. The prototype, PDIA1, has two redox-active CGHC domains that have four reactive Cys residues, two substrate binding domains, and a C-terminal ER retention sequence (KDEL) (Laurindo et al., 2012). Although PDIA1 is present mainly in the ER, it is also found in the cytosol (Parakh and Atkin, 2015; Turano et al., 2002; Wroblewski et al., 1992) and mitochondria (El Hindy et al., 2014) and on the cell surface (Soares Moretti and Martins Laurindo, 2017). Global PDIA1 knockout mice are embryonic lethal, and PDIA1 is dysregulated in neurodegenerative and cardiovascular diseases (Xu et al., 2014). PDIA1 has been shown to be involved in agonist-induced nicotinamide adenine dinucleotide phosphate (NADPH) oxidase (NOX) activation to increase ROS in vascular smooth muscle cells (VSMCs) (Laurindo et al., 2012). However, the role of PDIA1 in mitochondrial dynamics in ECs and postnatal angiogenesis *in vivo* in normal and pathological conditions such as diabetes remains unknown.

In the present study, we provide evidence that PDIA1 functions as a thiol reductase for Drp1 in ECs that maintains normal mitochondrial dynamics and endothelial function. Unexpectedly, loss of PDIA1 in ECs induces mitochondrial fragmentation and mtROS elevation without ER stress via increasing the sulfenylation of Drp1 at Cys^644^ and Drp1 activity, which drives EC senescence and impairs endothelium-dependent vasorelaxation and angiogenesis. *In vivo*, defective wound healing and angiogenesis in *PDIA1*^+/−^ mice or type 2 diabetes mice, which have reduced PDIA1 expression, are rescued by EC-targeted PDIA1 or Cys oxidation-defective mutant Drp1. Our findings indicate that restoring the endothelial PDIA1-Drp1 axis and/or targeting Drp1 Cys oxidation are important therapeutic strategies for treating diabetic vascular complications.

## RESULTS

### PDIA1Knockdown Induces Senescence and Endothelial Dysfunction

**Figure 1.**
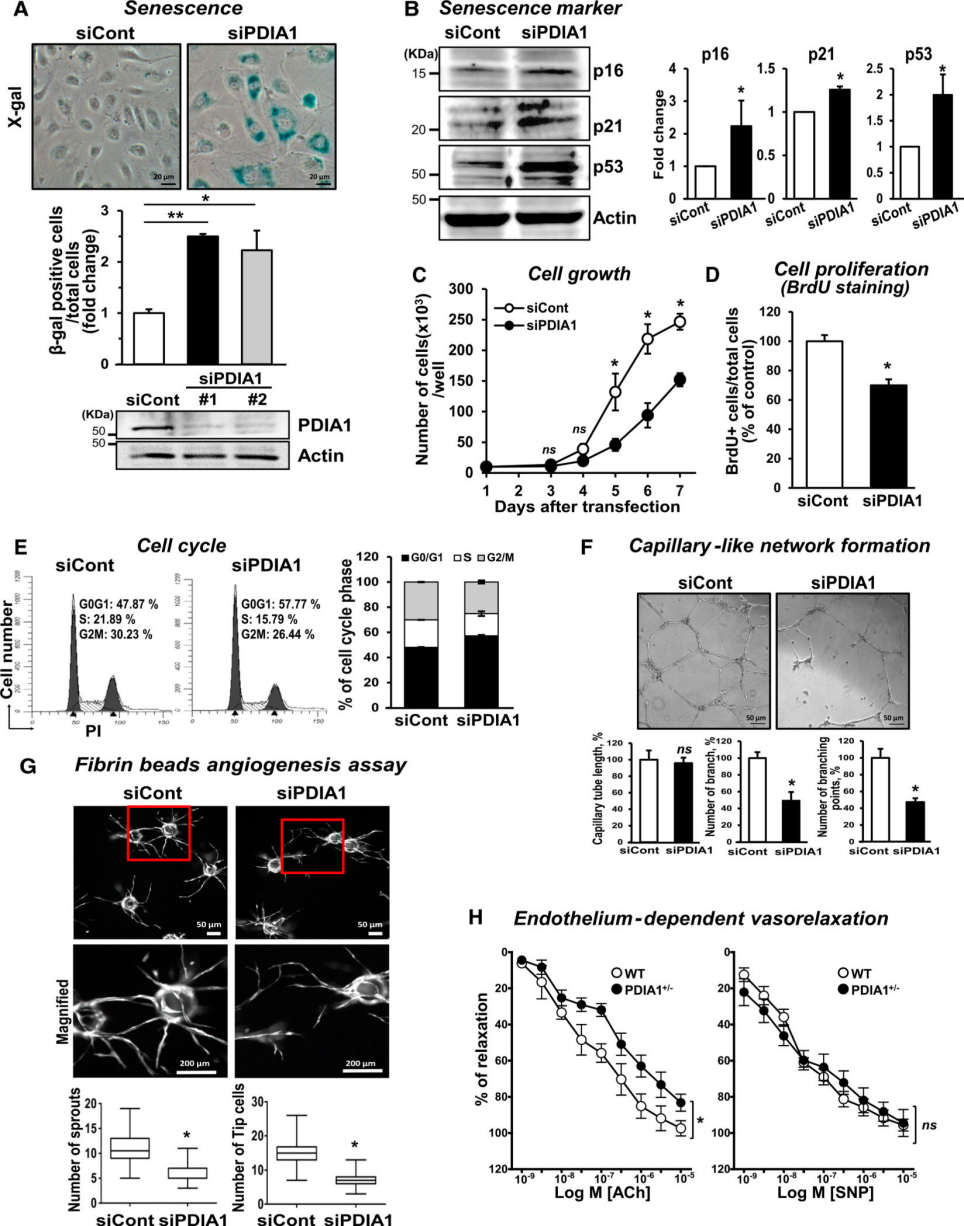
PDIA1 Knockdown Induces Senescence and Endothelial Dysfunction HUVECs were transfected with siRNAs for control (siCont) or PDIA1 (siPDIA1). (A) The two different PDIA1 siRNAs show senescence detected by β-galactosidase staining. (B) Protein expression for senescence marker proteins was detected by western blotting. (C) Cell growth was measured by cell counting. (D) Cell proliferation was determined by bromodeoxyuridine-positive (BrdU^+^) cells (percent) of total cells. (E) The cell cycle was measured using fluorescence-activated cell sorting (FACS) analysis. (F) Capillary-like network formation on Matrigel analyzed by the number of tube branches, branchpoints, or capillary tube length. (G) Capillary sprouting formation in the fibrin clot was analyzed by the number of sprouts and number of tip cells. (H) Acetylcholine (Ach)-induced endothelium-dependent and sodium nitroprusside (SNP)-induced endothelium-independent vasorelaxation in WT or *PDIA1*^+/−^ mice aortae precontracted with phenylephrine. Data are mean ± SEM (n = 3–9). *p < 0.05, **p < 0.001 versus siCont or WT. ns; non-significant.

To determine the function of endogenous PDIA1 in ECs, we examined the effect of PDIA1 knockdown using small interfering RNAs (siRNAs) in human umbilical vein endothelial cells (HUVECs). Silencing of PDIA1 induced significant morphological changes, such as round, flattened, or di- and/or multi-nucleustype cells (Figure S1A), and increased β-galactosidase activity at pH 6 (Figure 1A), demonstrating a senescence-like phenotype. This was further confirmed by increased expression of the senescence markers p16, p21, and p53 (Figure 1B) as well as decreased cell growth (Figure 1C), cell proliferation (Figure 1D), and induced cell cycle arrest at G0/G1 phase (Figure 1E) in PDIA1-depleted ECs compared with control siRNA-transfected ECs. We confirmed that PDIA1 knockdown did not alter the expression of other ER chaperons, such as ERp46, ERp5, or ERp72 (Figure S1C), showing the specificity of siPDIA1. PDIA1 knockdown efficiency showed until 5 days after siRNA transfection (Figure S1B).

Because endothelial senescence has been shown to contribute to endothelial dysfunction, we next examined the role of PDIA1 in angiogenesis and endothelium-dependent vasorelaxation (EDR). Silencing PDIA1 caused a significant decrease in capillary-like network formation on Matrigel in HUVECs (Figure 1F) or human aortic ECs (HAECs) (Figure S2E). Figure 1G shows that PDIA1 knockdown significantly reduced the number of capillary sprouts and tip cells per bead in a fibrin bead assay (Tung and Kitajewski, 2010), suggesting that PDIA1 is involved in capillary sprouting and branching morphogenesis *in vitro*. Figure 1H shows that PDIA1 haplodeficiency in mice (Figures S1D and S1E) caused impaired acetylcholine (ACh)-induced EDR without affecting endothelium-independent sodium nitroprusside (SNP)-induced vasodilation compared with wild-type (WT) mice. *PDIA1*^+/−^ mice showed increased senescence marker proteins, such as p16 and p53 (Figure S1F). These results suggest that PDIA1 is required to maintain endothelial function in isolated blood vessels.

### Silencing of PDIA1 Induces mtROS Elevation and Mitochondrial Dysfunction in ECs

**Figure 2.**
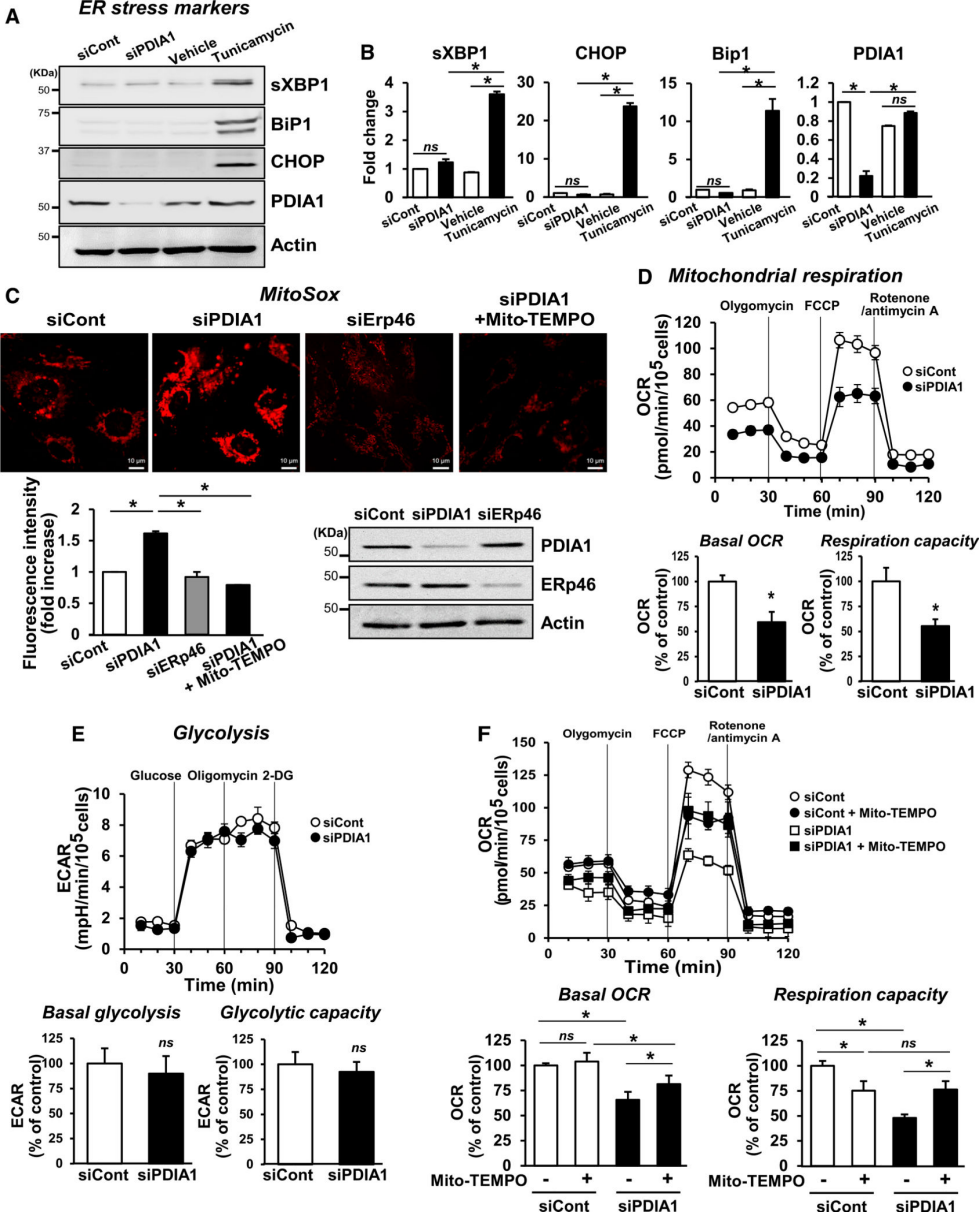
PDIA1 Knockdown Increases mtROS and Impairs Mitochondrial Respiratory Function without Inducing ER Stress in ECs (A–E) HUVECs were transfected with siCont or siPDIA1. (A and B) ER stress marker proteins (sXBP1, BiP1, or CHOP) were determined by western blotting (A) and their quantification was expressed as the fold change from control (siCont group, B). (C) Mitochondrial redox status (mtROS) detected by MitoSOX fluorescence, which is abolished by MitoTEMPO in siCont-, siPDIA1-, and siERp46-transfected ECs. Graphs represent the fold increase in fluorescence intensity from control (siCont group). Western blots show the protein knockdown efficiency for PDIA1 or ERp46. (D and E) Mitochondrial respiratory capacity (D) and glycolytic flux (E) measured by O_2_ consumption rate (OCR) and extracellular acidification rate (ECAR), respectively, using a Seahorse analyzer. (F) OCR measurement in ECs with or without MitoTEMPO treatment. Graphs represent the relative values normalized with the cell numbers, expressed as percent of control (siControl group). Data are mean ± SEM (n = 3–6). *p < 0.05 versus siCont or vehicle.

Given that the canonical function of PDIA1 is to assist protein folding in the ER (Wilkinson and Gilbert, 2004), we examined whether PDIA1 depletion-induced EC dysfunction is due to induction of ER stress. Surprisingly, PDIA1 knockdown using siRNA in ECs did not significantly increase ER stress marker proteins, including sXBP1, BiP1, or CHOP (Figures 2A and 2B), suggesting that ER stress does not explain the siPDIA1-induced EC phenotype. Because ROS play a role in cellular senescence, we next examined whether PDIA1 regulates ROS levels in ECs. Contrary to previous reports that PDIA1 is involved in agonistinduced NOX activation to increase ROS (Laurindo et al., 2012), we unexpectedly found that PDIA1 knockdown in resting HUVECs slightly increased the intracellular redox status detected by 2′,7′-dichlorodihydrofluorescein diacetate (DCF-DA) (Figure S2A), which was abolished by polyethylene glycol (PEG)-catalase (data not shown). Furthermore, PDIA1 knockdown dramatically increased the mitochondria redox status detected by MitoSOX fluorescence, which was abolished by MitoTEMPO, a scavenger of mitochondrially derived O_2_^−^ (Figure 2C) or by Mito-Tracker-CMTMRos fluorescence, which was abolished by overexpression of mitochondrially targeted catalase (mito-catalase), which scavenges mitochondrially derived H_2_O_2_ (Figure S2B) in HUVECs or HAECs (Figures S2F and S2G). These results suggest that PDIA1 knockdown increases mtROS levels without inducing ER stress in ECs. Of note, this response is specific to PDIA1 because silencing ERp46, another PDI family highly expressed in ECs, did not increase MitoSOX fluorescence (Figures 2C and S2B).

To further assess the role of PDIA1 in mitochondrial function, we examined mitochondrial respiration using an XF24 extracellular flux analyzer. PDIA1 knockdown in HUVECs significantly reduced the basal mitochondrial O_2_ consumption rate (OCR) and mitochondrial respiration capacity (Figure 2D) without affecting the extracellular acidification rate (ECAR), which measures glycolytic flux (Figure 2E). The reduced basal OCR and respiration capacity in PDIA1-depleted ECs were partially but significantly rescued by MitoTEMPO (Figure 2F). These findings suggest that loss of PDIA1 in ECs induces mitochondrial dysfunction in part through mtROS elevation, independent of ER stress or glycolysis.

### PDIA1 Knockdown Induces Mitochondrial Fragmentation in a PDI Redox Activity-Dependent Manner

**Figure 3.**
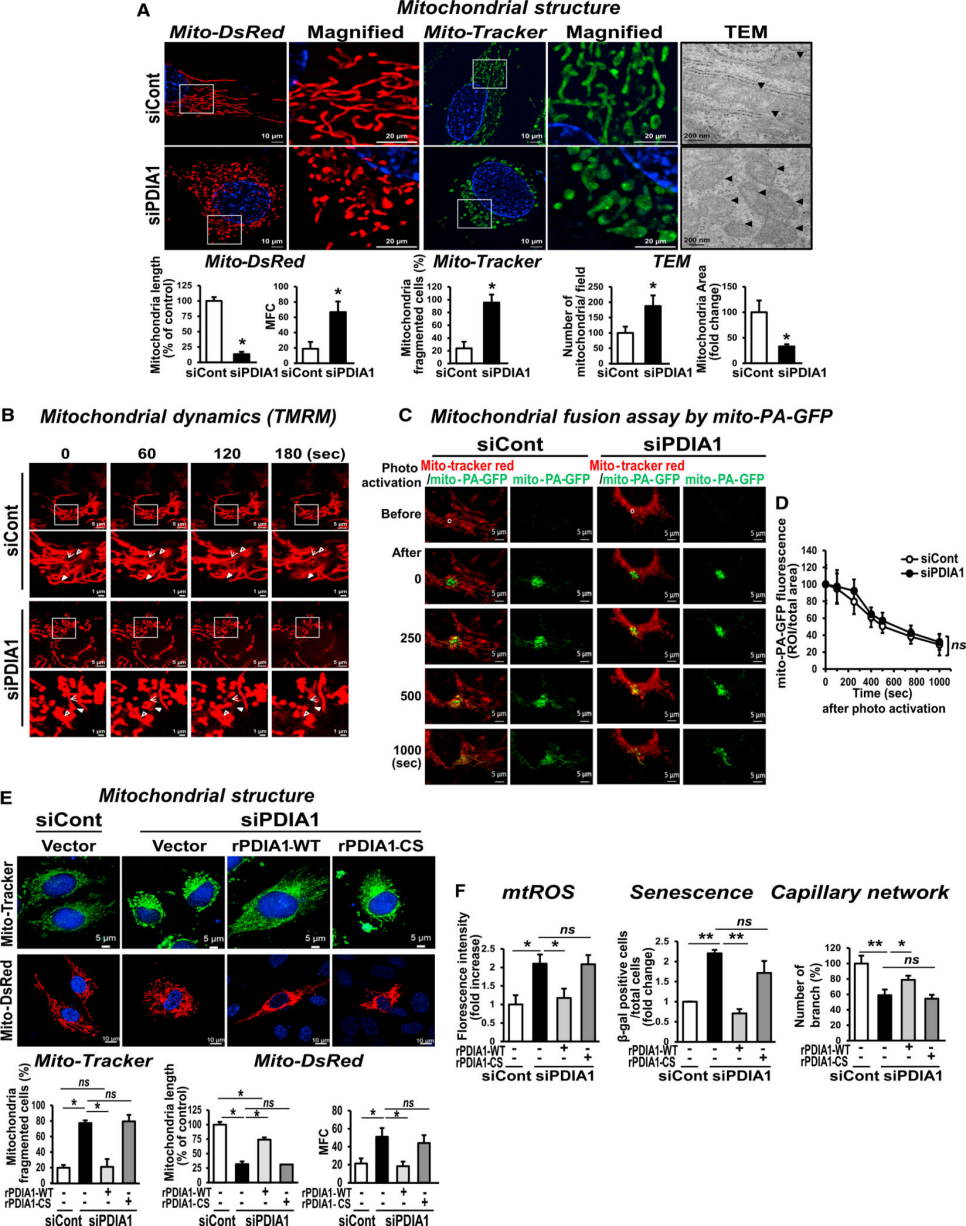
PDIA1 Knockdown Induces Mitochondrial Fragmentation and Endothelial Dysfunction in a Redox Activity-Dependent Manner in ECs (A and B) HUVECs were transfected with siCont or siPDIA1. (A) Mitochondrial morphology visualized by pMito-dsRed or Mito-Tracker Green using super-resolution microscopy (1003) as well as by transmission electron microscopy (TEM). Shown are quantification for mitochondrial length and mitochondrial fragmentation count (MFC) (for pMito-dsRed), mitochondrially fragmented cells (for Mito-Tracker), and the number of mitochondria and mitochondria area (for TEM). (B) Real-time imaging of mitochondrial dynamics visualized by the TMRM probe (50 nM) using confocal microscopy (100×). Each arrow shows a stable connection or dynamic disruption of the mitochondrial network in the siCont or siPDIA1 group, respectively. (C and D) PDIA1 depletion in ECs does not alter mitochondrial fusion. HUVECs transfected with siCont or siPDIA1 were infected with adenovirus (Ad).mito-PA-GFP and then incubated with Mito-Tracker Red. Live-cell imaging was performed before and after photoactivation of the minimum region (400 μm^2^, white circle) with a 405 nm laser using confocal microscopy. (C) On the left in each siCont and siPDIA1 group merged images are shown of Mito-Tracker Red (red) and mito-PA-GFP (green), whereas on the right in each group mito-PA-GFP is shown. (D) A decrease in the mean intensity of GFP fluorescence was measured as fusion. The objective magnification was 63× with 2× zoom. The representative images and averaged fluorescence intensities are from three independent experiments. (E and F) Ectopic expression of human siRNA-resistant FLAG-rat PDIA1 (rPDIA1-WT) or FLAG-rat PDIA1-CS (inactive mutant) in siCont- or siPDIA1-transfected HUVECs. (E) Mitochondrial morphology visualized by Mito-Tracker and Mito-dsRed was quantified by mitochondrially fragmented cells, mitochondrial length, and MFC, respectively. (F) Quantification of mitochondrial redox status (mtROS) measured by Mito-Tracker CMTMRos fluorescence, which was abolished by mito-catalase, senescence, and capillary-like network formation. Data are mean ± SEM (n = 3–5). *p < 0.05, **p < 0.001 versus siCont.

Because altered mitochondrial dynamics regulate mitochondrial function, we next examined the role of PDIA1 in mitochondrial morphology in ECs using Mito-dsRed to mark the entire mitochondrial network or Mito-Tracker Green, visualized by super-resolution confocal microscopy and transmission electron microscopy (TEM). Surprisingly, PDIA1 knockdown markedly increased mitochondrial fragmentation in HUVECs (Figures 3A and S2D) and HAECs (Figures S2F and S2G). To confirm this further, we performed real-time imaging of mitochondrial dynamics using a low concentration of tetramethylrhodamine methyl ester (TMRM) (50 nM) (Papanicolaou et al., 2011; Figure 3B). HUVECs transfected with control siRNA showed a stably connected mitochondrial network with regularly exchanged fission and fusion, whereas PDIA1-depleted ECs exhibited dynamic disruption of the mitochondrial network with a rapid increase in mitochondrial fragmentation (Videos S1 and S2).

To examine whether mitochondrial fragmentation induced in siPDIA1-treated ECs is caused by increased fission and/or decreased fusion, we examined mitochondrial fusion using mitochondrially targeted photoactivatable GFP (mito-PA-GFP). The time series images show that, in control siRNA (siCont)-treated ECs, the GFP signal spread out from the photoactivated region and decreased in intensity at later time points, indicating mitochondrial fusion (Figure 3C; Video S3). PDIA1-depleted ECs showed similar changes of mito-PA-GFP (Figure 3C; Video S4). Quantification of fluorescence intensity over time demonstrates that there is no significant difference in decrease of fluorescence in siCont- and siPDIA1-treated ECs (Figure 3D). This result suggests that PDIA1-depleted ECs activated mitochondrial fission but did not alter mitochondrial fusion.

To address the role of PDIA1 activity for regulating mitochondria fission, we performed rescue experiment by transfecting siRNA-resistant FLAG-ratPDIA1-WT (rPDIA1-WT) or FLAG-rPDIA1 catalytically inactive mutant (rPDIA1-CS), in which the two active site CGHC residues were mutated to SGHS residues (Hahm et al., 2013). We found that expression of rPDIA1-WT, but not rPDIA1-CS, rescued PDIA1 depletion-induced mitochondrial fragmentation (Figure 3E), mtROS, EC senescence, or impaired capillary network formation (Figure 3F). These results suggest that PDIA1 activity is required to maintain normal mitochondrial and EC functions.

### PDIA1 Knockdown Increases Mitochondrial Fragmentation and mtROS Levels by Increasing Drp1 Activity in ECs

**Figure 4.**
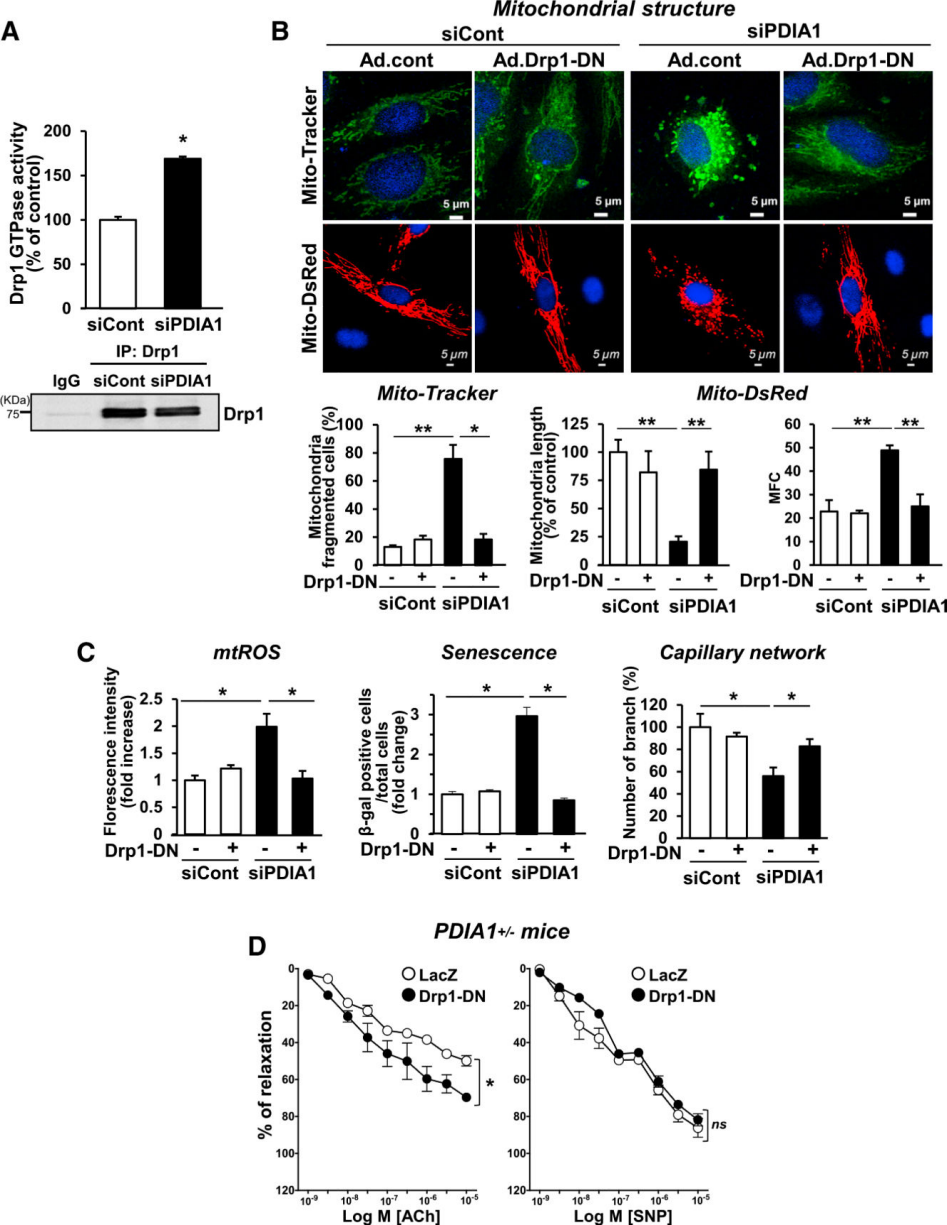
PDIA1 Knockdown Induces Endothelial Dysfunction by Increasing Drp1 Activity in ECs (A–C) HUVECs were transfected with siCont or siPDIA1.</P/>(A) Lysates immunoprecipitated with anti-Drp41 antibody were used to measure Drp1 GTPase enzyme activity. (B and C) Cells were infected with empty adenovirus (Ad.cont) or Ad.Drp1-K38A (Ad.Drp1-DN) and used to measure mitochondrial morphology visualized by Mito-Tracker and Mito-dsRed (B), mitochondrial redox status (mtROS) measured by Mito-Tracker CMTMRos, cell senescence, and capillary-like network formation (C). (D) ACh-induced endothelium-dependent and SNPinduced endothelium-independent vasorelaxation in *PDIA1*^+/−^ mice aorta transfected with Ad.LacZ (control) or Ad.Drp1-DN. Data are mean ± SEM (n = 3–4). *p < 0.05, **p < 0.001 versus siCont or Ad.cont.

Because Drp1 is a key regulator of mitochondrial fission (Elgass et al., 2013; Westermann, 2010), we next examined whether PDIA1 maintains mitochondrial dynamics via regulating Drp1 activity. We found that silencing PDIA1 in ECs significantly increased Drp1 GTPase activity (Figure 4A) and that overexpression of Drp1-dominant-negative (DN) (Drp1-K38A) (Figures 4B, 4C, and S4C), or Mdivi-1, a specific Drp1 inhibitor (Cassidy-Stone et al., 2008; Figures S4A and S4B) rescued siPDIA1-induced mitochondrial fragmentation, mtROS, senescence, and impaired capillary network formation. Moreover, the impaired EDR in *PDIA1*^+/−^ aorta was rescued by gene transfer of Drp1-DN (Figures 4D and S4D). These results suggest that PDIA1 knockdown-induced Drp1 activity promotes mitochondrial fragmentation, enhancing mtROS elevation and EC dysfunction.

### PDIA1 Knockdown Induces Sulfenylation of Drp1 at Cys^644^, Increasing Mitochondrial Fragmentation, mtROS, and EC Dysfunction

**Figure 5.**
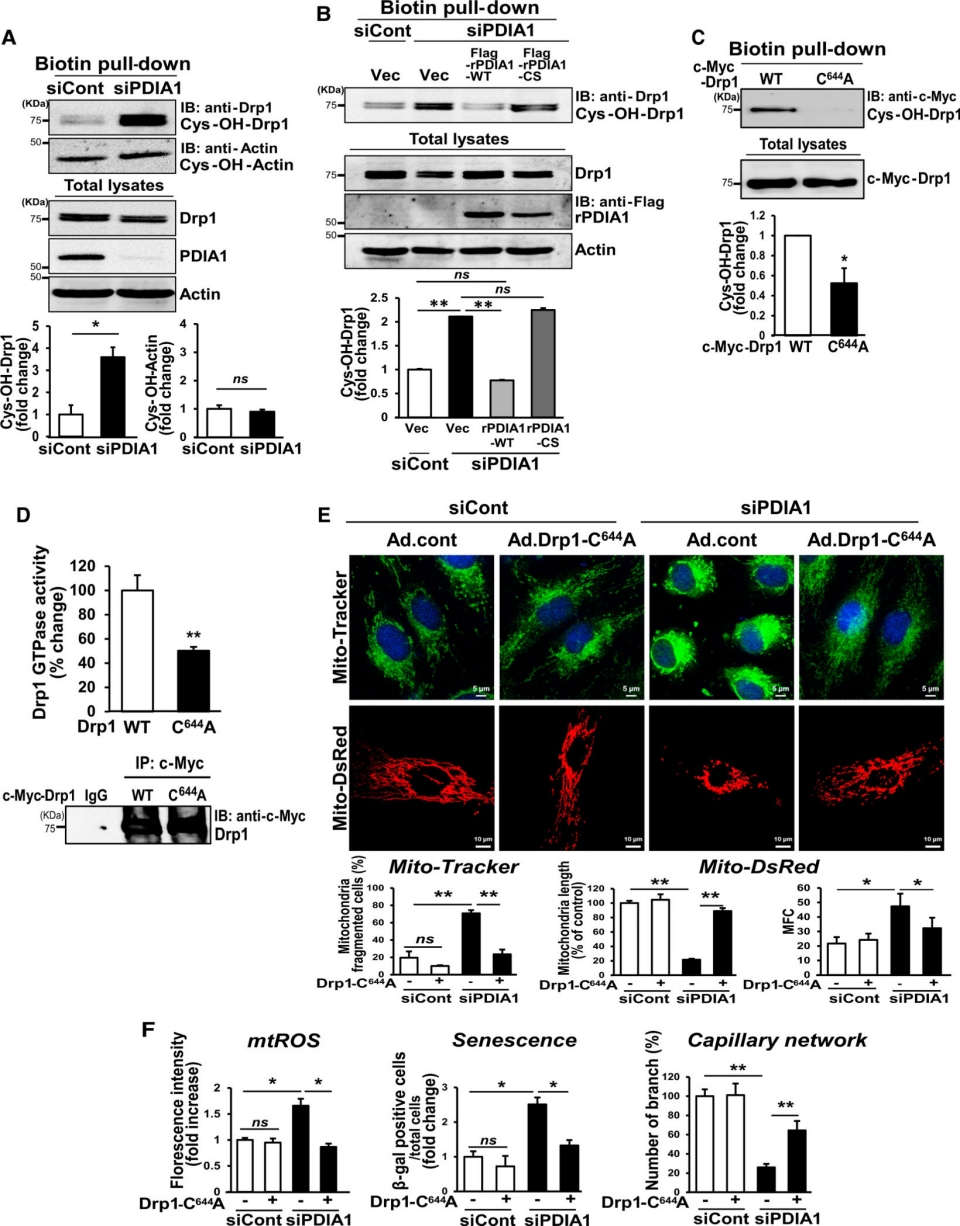
PDIA1 Knockdown Increases Drp1 Cys^644^ Oxidation and GTPase Activity in ECs (A and B) HUVECs were transfected with siCont or siPDIA1. (A) DCP-Bio1-labeled lysates were pulled down with streptavidin beads and then immunoblotted with Drp1 or actin antibody to detect their Cys-OH formation. (B) Cells were also transfected with FLAG empty vector, FLAG-rPDIA1-WT, or FLAG-rPDIA1-CS (inactive form) and used to measure Cys-OH formation of Drp1 as in (A). (C and D) PDIA1 siRNA-transfected HUVECs were infected with Ad expressing c-Myc-Drp1-WT or c-Myc-Drp1-C^644^A. (C) DCP-Bio1-labeled lysates were pulled down with streptavidin beads and immunoblotted with c-Myc antibody to measure Cys-OH-formed c-Myc-Drp1. (D) Cells immunoprecipitated with anti-c-Myc antibody were used to measure Drp1 GTPase activity. (E and F) PDIA1 siRNA-transfected HUVECs infected with Ad.cont or Ad.Drp1-C^644^A. (E) Cells were used to measure mitochondrial morphology visualized by Mito-Tracker (top) and Mito-dsRed (bottom) and quantified (percent) for mitochondrially fragmented cells, mitochondrial length, and MFC, respectively. (F) Quantification of mitochondrial redox status (mtROS) measured by Mito-Tracker CMTMRos, senescence, and capillary-like network formation. Data are mean ± SEM (n = 3–4). *p < 0.05, **p < 0.001.

We next investigated how loss of PDIA1 increases Drp1 GTPase activity, which has been shown to be regulated by various PTMs, such as phosphorylation. We found that PDIA1 depletion had no significant effects on Drp1 phosphorylation at Ser^616^ and Ser^637^ or expression of mitochondrial fusion proteins while decreasing Drp1 protein expression (Figures S5A and S5B). Because PDIA1 functions as a thiol oxidoreductase, we examined whether Drp1 is oxidized in PDIA1-depleted ECs using a biotin-conjugated Cys-OH trapping probe, DCP-Bio1 (Kaplan et al., 2011; Poole and Nelson, 2008). PDIA1 knockdown in HUVECs significantly increased Cys-OH formation (sulfenylation) of Drp1 without altering that of actin (Figure 5A), which was rescued by re-expressing rPDIA1-WT but not rPDIA1-CS (Figure 5B). Because Cys^644^ of Drp1 is a key redox-sensitive Cys residue (Cho et al., 2009), we next examined the role of Cys^644^ in Drp1 Cys oxidation. PDIA1 knockdown-induced sulfenylation of cMyc-Drp1-WT (Figure 5C) or endogenous Drp1 (Figure S5D), GTPase activity (Figure 5D) and its multimer formation in non-reducing gels (data not shown) were significantly inhibited by overexpression of Drp1-C^644^A (Figure S5E). Moreover, siPDIA1-induced phenotypes, such as mitochondrial fragmentation (Figures 5E) as well as mtROS elevation, and EC dysfunction, such as senescence or impaired capillary formation (Figure 5F), were rescued by overexpression of Drp1-C^644^A. These results suggest that Drp1 sulfenylation at Cys^644^ induced by loss of PDIA1 reductase activity increases mitochondrial fragmentation, promoting mtROS elevation and EC dysfunction. We also examined the possible role of mtROS and found that mito-catalase overexpression (Figures S3A and S3B) or MitoTEMPO (Figures S3C and S5C) prevented siPDIA1-induced mitochondrial fragmentation or Drp1 sulfenylation. Thus, it seems that there is a positive feedforward mechanism through which mtROS induced by the Drp1-CysOH-mitochondria fragmentation axis increases Drp1 sulfenylation, which further enhances mitochondrial fission.

### PDIA1 Binds to Drp1 to Reduce Its Redox Status and Activity

**Figure 6.**
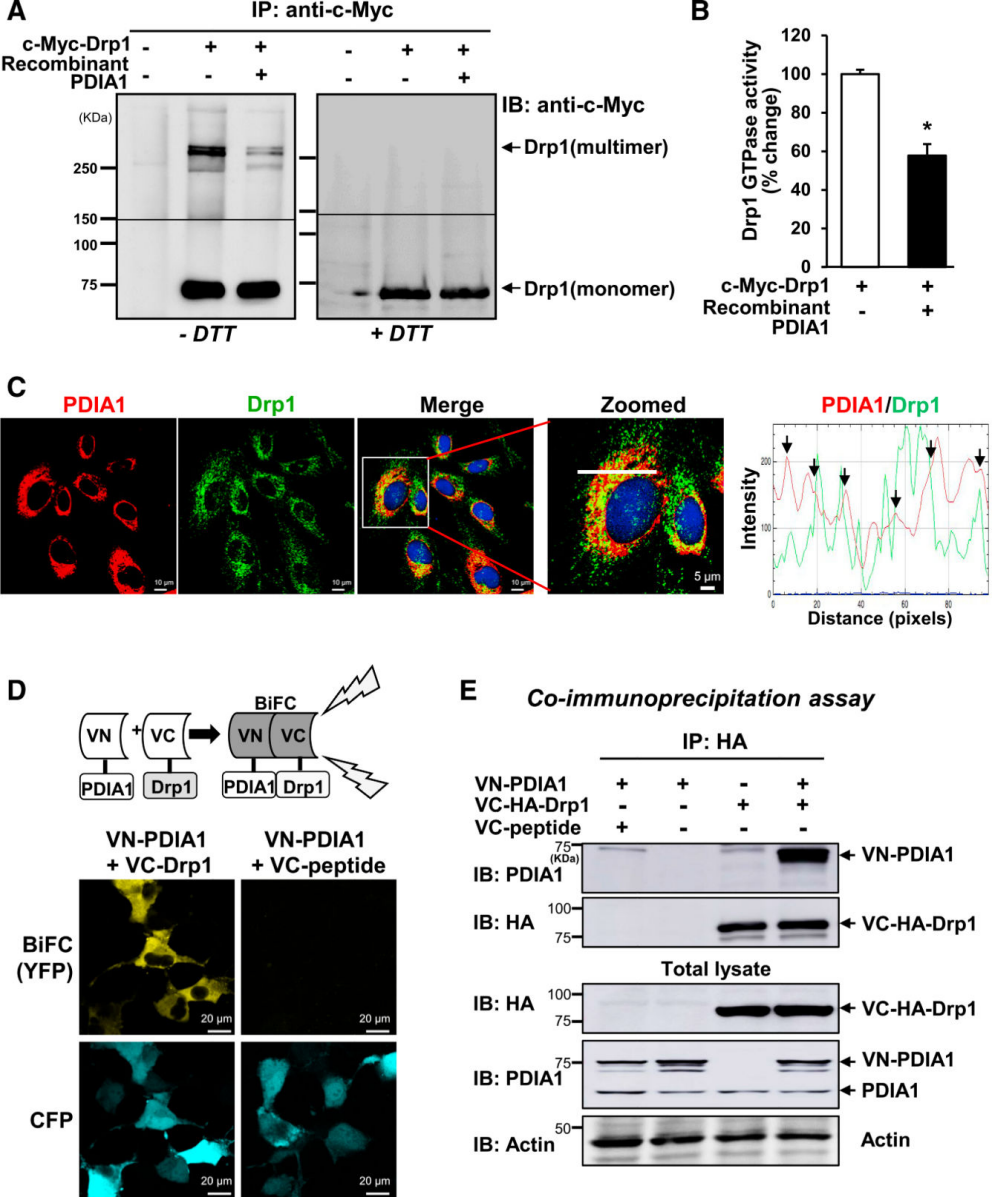
PDIA1 Binds to Drp1 and Reduces GTPase Activity, (A and B) HUVECs infected with Ad.cont or Ad. c-Myc-Drp1-WT were immunoprecipitated with c-Myc antibody, and lysates were incubated with or without human recombinant PDIA1 protein. Mixtures were subjected to SDS-PAGE under nonreducing (−DTT) and reducing (+DTT) conditions and used for immunoblotting with anti-c-Myc antibody to measure the redox status of c-Myc-Drp1 (A) or Drp1 GTPase activity (B). Data are shown as mean ± SEM (n = 3–5). *p < 0.05. (C) Co-localization of PDIA1 and Drp1 in HUVECs, showing yellow fluorescence in merged images, was analyzed by comparing the fluorescence intensity for each protein (white line on enlarged image). (D) BiFC assay. Co-transfection of N-terminal Venus-PDIA1 (VN-PDIA1) + C-terminal Venus-HADrp1 (VC-Drp1) + protein expressing enhanced cyanide fluorescent protein (pECFP) (transfection positive control) and VN-PDIA1 + C-terminal Venus-negative peptide (VC-peptide) + pECFP in Cos1 cells. The YFP signal shows interaction of PDIA1 and Drp1. (E) HEK293T cells co-transfected with the Venus vectors described in (D) were immunoprecipitated with HA antibody, and immunoprecipitation (IP) or non-IP lysates were separated by SDS-PAGE, followed by immunoblotting with the indicated antibodies. Shown are representative images or blots from 3–4 different experiments.

We then examined whether PDIA1 directly regulates the Drp1 redox status and its GTPase activity. Figure 6A shows that recombinant PDIA1 reduces oligomerized and oxidized Drp1 in c-Myc-Drp1 immunoprecipitates in HUVECs transfected with c-Myc-Drp1 in non-reducing gels. In parallel, Drp1 GTPase activity in c-Myc-Drp1 immunoprecipitates was significantly inhibited by recombinant PDIA1 (Figure 6B). These results suggest that PDIA1 reduces the Drp1 redox status to inhibit its GTPase activity. Next, to determine the mechanism by which PDIA1 regulates Drp1 activity, we examined whether PDIA1 binds to Drp1 and found that PDIA1 colocalizes with Drp1 in HUVECs at the perinucleus and in the cytosol (Figure 6C). Moreover, a BiFC (bimolecular fluorescence complementation) assay shows that yellow fluorescence protein (YFP) fluorescence was increased by co-transfection of the Venus-N-terminal vector expressing PDIA1 (VN-PDIA1) and the Venus-C-terminal vector expressing Drp1 (VC-Drp1) but not VC-expressing scrambled peptide (negative control) (Figure 6D). In addition, we confirmed that VN-PDIA1 associates with VC-Drp1 by co-immunoprecipitation analysis (Figure 6E).

### EC-Specific PDIA1 Gene Transfer Rescues Impaired Wound Healing in Type 2 Diabetes Mice

**Figure 7.**
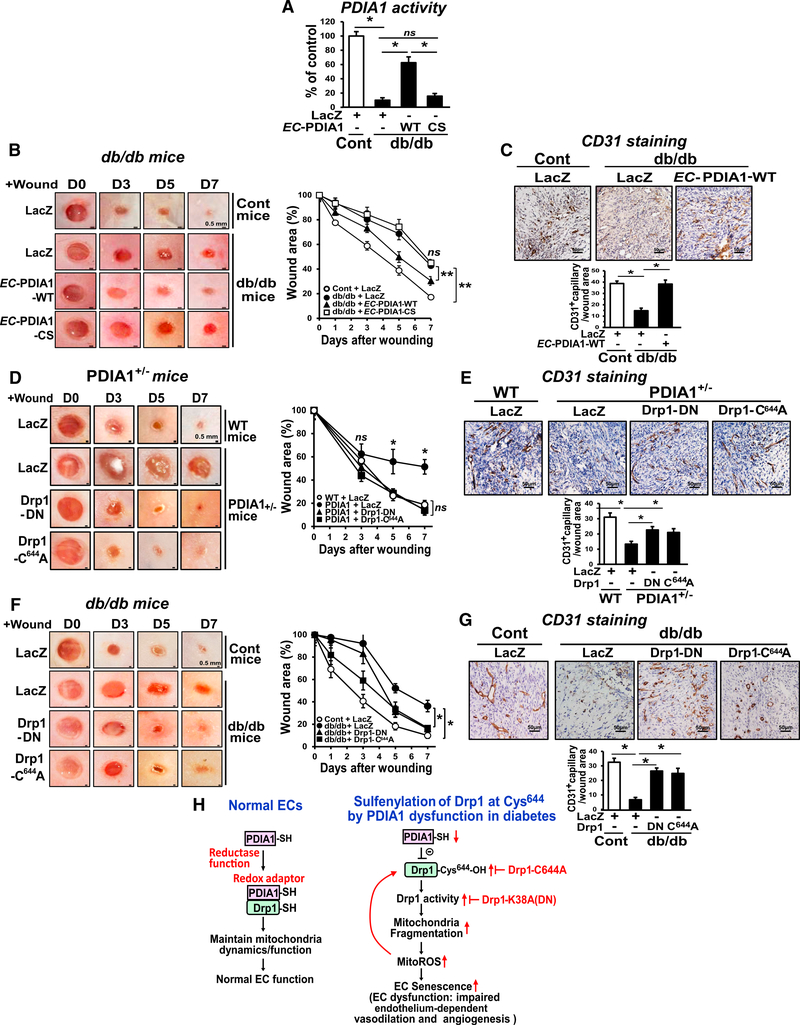
EC-Specific PDIA1, Drp1-DN, or Drp1-C^644^A Mutant Gene Transfer Rescues Impaired Wound Healing in T2DM or *PDIA1*^+/−^ Mice (A–C) Control (Cont) and db/db mice were wounded on the back skin, and wound regions were overlaid with Ad.LacZ, Ad.EC-PDIA1-WT (PDIA1 with the VE-cadherin promoter), or Ad.EC-PDIA1-CS, and then the wound closing rate was measured for 7 days. The wounded skin tissues were used to analyze PDIA1 enzyme activity (A) or wound area, expressed as percent of that measured right after the wounding (B), or CD31^+^ cells using CD31 antibody (C). (D–G) Ad.LacZ, Ad.Drp1-DN, or Ad.Drp1-C^644^A was overlaid on the wound regions in WT or *PDIA1*^+/−^ (D) or control or db/db mice (F), and then the wound closing rate was measured for 11 days. (E and G) Wounded skin tissues were used to measure CD31^+^ cells. Data are mean ± SEM (n = 3). *p < 0.05. (H) Proposed model showing that PDIA1 binds to Drp1 in the cytosol and functions as a thiol reductase for Drp1 to keep it in a reduced and inactive state, maintaining normal mitochondrial dynamics and EC function in resting ECs. Under pathological conditions such as diabetes, PDIA1 dysfunction increases sulfenylation of Drp1 and activity, enhancing mitochondrial fragmentation, which drives mtROS elevation and endothelial senescence and dysfunction.

We then examined the functional role of PDIA1 *in vivo* using a mouse skin wound healing model with type 2 diabetes mellitus (T2DM), in which endothelial cell senescence plays a role in its pathogenesis (Palmer et al., 2015). We found that PDIA1 protein expression was markedly downregulated in skins of db/db mice compared with control mice (Figures S6A and S6B). Gene transfer of PDIA1-WT with EC-specific vascular endothelial (VE)-cadherin promoter (EC-PDIA1-WT), but not inactive EC-PDIA1-CS, in wound sites of db/db mice rescued reduced PDIA1 protein expression (Figure S6B) and activity (Figure 7A) as well as wound healing (Figure 7B). We confirmed that CD31^+^ capillary density was decreased in db/db mice, which was restored by EC-PDIA1-WT gene transfer (Figures 7C and S6C), and that EC-PDIA1-WT was specifically expressed in ECs (Figure S7A). We also verified that similar results were obtained with high-fat diet-induced T2DM mice (Figures S7B–S7D).

### Drp1-DN or Drp1-C^644^A Mutant Gene Transfer Rescues Impaired Wound Healing in T2DM or *PDIA1*^+/−^ Mice

To gain insight into the role of the PDIA1-Drp1 axis in wound healing, we used *PDIA1*^+/−^ and db/db mice. We found that wound healing was significantly impaired in *PDIA1*^+/−^ mice (Figure 7D) and db/db mice (Figure 7F) compared with control mice injected with β-galactosidase (LacZ). However, gene transfer of Drp1-DN or Drp1-C^644^A to the wounded tissue significantly restored blunted wound healing and CD31^+^ capillary density in *PDIA1*^+/−^ mice (Figures 7E and S6D). We then examined the role of Drp1 in impaired wound healing in T2DM mice and found that gene transfer of Drp1-DN or Drp1-C^644^A in wounded tissues of db/db mice rescued impaired wound healing and angiogenesis (CD31^+^ capillary density) compared with LacZ (Figures 7F and 7G and S6E). These results suggest that targeting the PDIA1-Drp1 axis is an important therapeutic strategy for restoring impaired wound repair in T2DM.

## DISCUSSION

How redox signals organize coupling mitochondrial dynamics/ morphology to EC function has remained unclear. In this study, we provide a mechanism of redox regulation of mitochondrial fission via PDIA1 that functions as a thiol reductase for Drp1, which maintains healthy mitochondrial and endothelial function. Here we show that: (1) PDIA1 depletion in human ECs drives senescence or impaired endothelium-dependent vasodilation or angiogenesis without inducing ER stress; (2) these PDIA1 knockdown-induced EC phenotypes are rescued by expression of PDIA1-WT or Drp1-DN, which prevents mitochondrial fission, but not by the inactive PDIA1-CS mutant; (3) mechanistically, PDIA1 associates with Drp1 to reduce its redox status, whereas loss of PDIA1 increases sulfenylation of Drp1 at Cys^644^ and Drp1 activity, promoting mitochondrial fragmentation and mtROS, leading to endothelial dysfunction; and (4) defective wound healing and angiogenesis in T2DM mice, which have reduced PDIA1 expression, or in *PDIA1*^+/−^ mice are rescued by gene transfer of EC-targeted PDIA1 or Drp1 DN or Drp1 Cys^644^A Vutant. These results suggest that sulfenylation of Drp1 at Cys^644^ induced by dysregulation of PDIA1 links excessive mitochondrial fission to EC senescence, involved in aging-associated vascular diseases such as diabetes (Figure 7H).

A loss-of-function approach and rescue experiments with siRNA-resistant PDIA1-WT or inactive PDIA1-CS mutant reveal that endogenous PDIA1 redox activity is required to protect against EC senescence. Because PDIs are known to facilitate the proper folding of nascent proteins in the ER (Eletto et al., 2014), it is generally considered that PDIs prevent ER stress in neurodegenerative and other diseases (Walker et al., 2010). However, we found that PDIA1 depletion promotes ECs dysfunction, including EC senescence or impaired endothelium-dependent vasodilation or angiogenesis without inducing ER stress. This may be due to the presence of other ER chaperone proteins and PDI families that are insensitive to siPDIA1. Consistent with our result, Rutkevich et al. (2010) reported that depletion of PDI or other PDI family members does not increase unfolded protein responses. Thus, ER stress induction by loss of PDI function might be cell type- and context-specific and depends on expression of other PDI families and ER chaperones (Laurindo et al., 2014).

Our study demonstrates that PDIA1 depletion in human ECs increases fragmentation of mitochondria and mtROS, inducing endothelial dysfunction, such as EC senescence and impaired EDR and angiogenesis. Real-time imaging of mitochondrial dynamics using a low concentration of TMRM and mito-PAGFP reveals that PDIA1-depleted ECs activate mitochondrial fission without altering mitochondrial fusion. Of note, the siPDIA1-induced increase in mtROS is unexpected because PDIA1 has been shown to be involved in agonist-induced NOX activation in VSMCs and macrophages (Laurindo et al., 2012). Camargo et al. (2013) reported that PDIA1 or the PDI family member ERp46 mediates cytokine tumor necrosis factor alpha (TNF-α)-induced NOX activation in ECs. By contrast, we found that PDIA1 depletion in unstimulated ECs slightly increases cytosolic H_2_O_2_, which may be diffused from the mitochondria. Furthermore, silencing of ERp46 in ECs does not increase mtROS at the basal state, suggesting that the effects on mtROS and mitochondrial function are specific to PDIA1.

The next question is how PDIA1 knockdown increases Drp1 GTPase activity, which is the key response to induce mitochondrial fragmentation and EC senescence. It has been shown that Drp1 GTPase activity is regulated by various PTMs, such as phosphorylation (Elgass et al., 2013; Westermann, 2010); however, we found that Drp1-pSer^616^ or Drp1-pSer^637^, which increases or decreases Drp1 activity, respectively, is not altered, whereas total Drp1 protein is rather decreased by PDIA1 knockdown in ECs. Sulfenylation (Cys-OH formation) is a reversible initial step in ROS-mediated oxidation of reactive Cys residues of proteins (Paulsen and Carroll, 2010; Poole and Nelson, 2008). Given that Drp1 has a reactive Cys residue, Cys^644^ (Cho et al., 2009), we examined whether Drp1 is oxidized in PDIA1-depleted ECs. Experiments using a biotin-labeled Cys-OH trapping probe and Drp1 Cys oxidation-defective mutant, Drp1-C^644^A, reveal that loss of PDIA1 increases Drp1 sulfenylation at Cys^644^, promoting Drp1 GTPase activity and mitochondrial fragmentation, which drives mtROS elevation and EC dysfunction. Of note, NO has been shown to induce a fragmented mitochondrial phenotype through Drp1-SNO formation via increasing Drp1 activity in neurodegenerative diseases (Cho et al., 2009). However, this evidence seems to be controversial because Bossy et al. (2010) reported that Drp1-SNO does not increase GTPase activity. Thus, our evidence for sulfenylation of Drp1 at Cys^644^ may implicate a new mechanism for redox regulation of Drp1 activity and mitochondrial fission. In this study, Drp1-C^644^A may act as a dominant-negative mutant and, thus, compete with endogenous sulfenylated Drp1, limiting mitochondrial fragmentation in PDIA1-depleted ECs. However, we cannot eliminate the possibility that mutation of Cys^644^ may induce a conformational change to regulate Drp1 activity independent of inhibiting Cys oxidation. Furthermore, PDIA1 may induce Cys oxidation at other sites or PTM of Drp1, such as S-glutathionylation, sumoylation, ubiquitination, and acetylation (Elgass et al., 2013) or regulate other fission and fusion proteins.

Although it is difficult to demonstrate the causality between mitochondrial fission and mtROS elevation induced by PDIA1 knockdown, it has been reported that mitochondrial fragmentation increases ROS (Yu et al., 2006) or mtROS levels (Kluge et al., 2013; Shenouda et al., 2011) in pathological conditions such as diabetes. Consistently, we show that loss of PDIA1-induced mitochondrial fragmentation and mtROS as well as defective EC phenotypes are rescued by Drp1-DN or the Drp1 inhibitor Mdivi1 (Cassidy-Stone et al., 2008). These results support the notion that Drp1-mediated mitochondrial fragmentation is causal for mtROS elevation and EC dysfunction in PDIA1-depleted ECs. However, we also found that reducing mtROS inhibits mitochondrial fragmentation, respiratory deficit, or Drp1-SOH formation induced by PDIA1 knockdown. Taken together, there seems to be a positive feedforward mechanism by which Drp1 sulfenylation promotes mitochondrial fragmentation and mtROS, which, in turn, further enhances Drp1 sulfenylation, leading to EC senescence (Figure 7H). Moreover, we cannot exclude the possibility that mtROS elevation is the proximal event for inducing Drp1 sulfenylation.

Evidence suggests that PDI functions as a redox adaptor and organizer (Soares Moretti and Martins Laurindo, 2017) and that the majority of PDI in quiescent ECs is in a reduced form and functions as a thiol reductase (Wilkinson and Gilbert, 2004). In line with this notion, we show that PDIA1 binds to Drp1 to reduce its redox status, inhibiting GTPase activity and mitochondria fragmentation, which maintains normal mitochondrial dynamics and EC function. To keep Drp1 and PDIA1 in the reduced state, intracellular glutathione (GSH) levels may also play an important role. It has been shown that GSH, its oxidation to glutathione disulfide (GSSG), and the consequent change in the GSH/GSSG ratio regulate mitochondrial morphology (Costa et al., 2003; Schafer and Buettner, 2001). Shutt et al. (2012) reported that GSSG induces mitochondrial fusion via formation of disulfide bond-mediated mitofusin oligomers, which protects against stress-induced apoptosis and mitophagy. In contrast, our study demonstrates that PDIA1 functions as a reductase for Drp1 and that PDIA1 depletion-induced sulfenylation of Drp1 promotes the formation of disulfide-mediated Drp1 multimers and consequent mitochondrial fragmentation, which drives mtROS elevation and EC senescence. The relationship between redox-dependent modulation of mitochondrial fission and fusion should be clarified in a future study.

Based on our co-localization and BiFC assays as well as previous reports showing that inactive Drp1 is mainly localized in the cytosol (Chang and Blackstone, 2010), it is likely that PDIA1 associates with Drp1 in the cytosol to reduce its redox status. Although the cytoplasmic localization of PDIA1 remains controversial (Soares Moretti and Martins Laurindo, 2017), cell fractionation studies demonstrate that PDI is localized in the cytosol in resting and activated ECs (Jasuja et al., 2010). PDIA1 has been shown to interact with cytosolic proteins, such as SOD1 (Atkin et al., 2017), guanylate cyclase (Heckler et al., 2013), RhoA or RhoGDI (Laurindo et al., 2012; Pescatore et al., 2012), p47phox (de A Paes et al., 2011), Rac1 (Pescatore et al., 2012), or β-actin Cys^374^ (Sobierajska et al., 2014), to regulate their activity and/or redox status. Although it is unclear how PDIA1 can localize in the cytosol, it is possible that a part of total PDIA1 localized at the ER may be released into the cytosol and that PDIA1 secreted from ECs may reenter the cytosol, thereby reducing Drp1. Furthermore, endothelial surface PDIA1 has been shown to promote thrombus formation (Jasuja et al., 2010). However, we found that neutralizing the anti-PDIA1 antibody does not induce mitochondrial fission or senescence in ECs (unpublished data), eliminating the role of cell-surface PDIA1 in regulating mitochondrial dynamics and function.

The clinical implication of this study is related to the fact that, in pathological conditions such as diabetes, mitochondrial fragmentation has been shown to contribute to endothelial dysfunction (Kluge et al., 2013; Shenouda et al., 2011). However, the underlying mechanisms and functional significance *in vivo* were unknown. The present study shows that defective wound healing in T2DM mice, which have reduced PDIA1 expression, or *PDIA1*^+/−^ mice is rescued by gene transfer of EC-targeted PDIA1, Drp1 DN, or Drp1 Cys^644^ mutant. Thus, enhancing endothelial PDIA1 function or inhibiting Drp1 Cys oxidation and activity restores impaired angiogenesis in diabetic vascular complications (Figure 7H). Addressing the mechanism by which PDIA1 is downregulated in diabetic tissues is the beyond the scope of this study. In contrast, platelet-specific PDIA1 knockout mice show that platelet extracellular PDIA1 is involved in injury-induced thrombus formation (Kim et al., 2013). Thus, PDIA1 function becomes protective or pathological, depending on the cell type, subcellular localization, redox environment, or disease model. In addition to expression level, PTM of PDI mediated through redox modification such as S-gluthathionylation (Townsend et al., 2009), S-nitrosylation (Uehara et al., 2006), or sulfenylation (Kenche et al., 2016) may regulate PDI activity and function.

In summary, our findings demonstrate a link between PDIA1 and Drp1 oxidoreduction, which protects against excess mitochondrial fission and ROS elevation, leading to endothelial dysfunction in diabetic vascular complications (Figure 7H). These findings provide insights into restoring the endothelial PDIA1-Drp1 axis and/or targeting Cys oxidation of key proteins regulating mitochondrial dynamics and functions as attractive therapeutic strategies for various diseases associated with endothelial senescence, such as diabetes, atherosclerosis, and aging-related disorders.

## EXPERIMENTAL PROCEDURES

### Cell Culture

Primary HUVECs were cultured in EndoGRO (EMD Millipore) with 5% fetal bovine serum (FBS) and used for experiments until passage 6.

### Fibrin Bead Angiogenesis Assay

HUVECs infected with lenti-red fluorescence protein (RFP) were used for a 3D fibrin bead assay to analyze capillary tube formation and sprouting, as reported previously (Tung and Kitajewski, 2010).

### Mitochondrial Structure Analysis

The Mito-dsRed vector with the mitochondrial targeting sequence and Mito-Tracker Green FM (Invitrogen) were used to visualize and analyze mitochondrial structure. Images were taken by super-resolution microscopy (Nikon) or confocal microscopy (Zeiss LSM710). For live-cell imaging of mitochondria dynamics, 50 nM TMRM (Invitrogen) was used (Papanicolaou et al., 2011). For mitochondrial fusion live-cell imaging, mito-PA-GFP was used as reported previously (Rehman et al., 2012).

### DCP-Bio1 Assay to Detect Sulfenylated Proteins

HUVECs were lysed in the presence of 200 μM DCP-Bio1 (KaraFast, USA, and Dr. Leslie Poole at Wake Forest University), and DCP-Bio1-bound sulfenylated proteins were pulled down with streptavidin beads, followed by immunoblotting, as we reported previously (Kaplan et al., 2011).

### BiFC Assay

COS1 cells were transfected with N-terminal Venus-PDIA1 and C-terminal Venus-Drp1 or negative control peptide. The positive YFP signals showed interaction between PDIA1 and Drp1, as reported previously (Okur et al., 2014).

### Animals

All animal studies followed protocols approved by the Animal Care and Institutional Biosafety Committee of the University of Illinois at Chicago and the Medical College of Georgia at Augusta University. Mice were used at 8–12 weeks for C57Bl6 (WT) and *PDIA1*^+/−^ mice (Hahm et al., 2013) or 13–16 weeks for db/db T2DM mice and C57Bl6 (control) mice. Both males and females were used.

### Mouse Wound Healing Angiogenesis Model

The backside of the skin of mice was wounded using a 3-mm punch, and the wound closure rate was measured. To evaluate rescue effects, adenoviruses expressing EC-specific PDIA1-WT, Drp1-DN, Drp1-C^644^A, LacZ (control) were overlaid on the wounded region of db/db mice, and the wound closure rate was measured.

### Statistical Analysis

Statistical significance was assessed by two-tailed paired/unpaired Student’s t test or ANOVA on untransformed data, followed by comparison of group averages by contrast analysis using the Super ANOVA statistical program (Abacus Concepts, Berkeley, CA). p < 0.05 was considered significant.

## Supplementary Material

Movie S1

Movie S2

Movie S3

Movie S4

Supplemental file

## Highlights

- PDI functions as a thiol reductase for mitochondrial fission protein Drp1
- Loss of PDI induces Drp1 sulfenylation at Cys^644^, driving endothelial senescence
- Dysfunction of endothelial PDI contributes to impaired wound healing in diabetes
- Restoring endothelial PDI-Drp1 axis protects against diabetic vascular complication
