# Supplementary material for: Redox Regulation of Mitochondrial Fission Protein Drp1 by Protein Disulfide Isomerase Limits Endothelial Senescence
Source: Cell Rep. Author manuscript; Available in PMC 2019 Jan 8. (PMC6324937; doi:10.1016/j.celrep.2018.05.054)
Supplement: Supplemental file [file NIHMS993103-supplement-Supplemental_file.pdf]

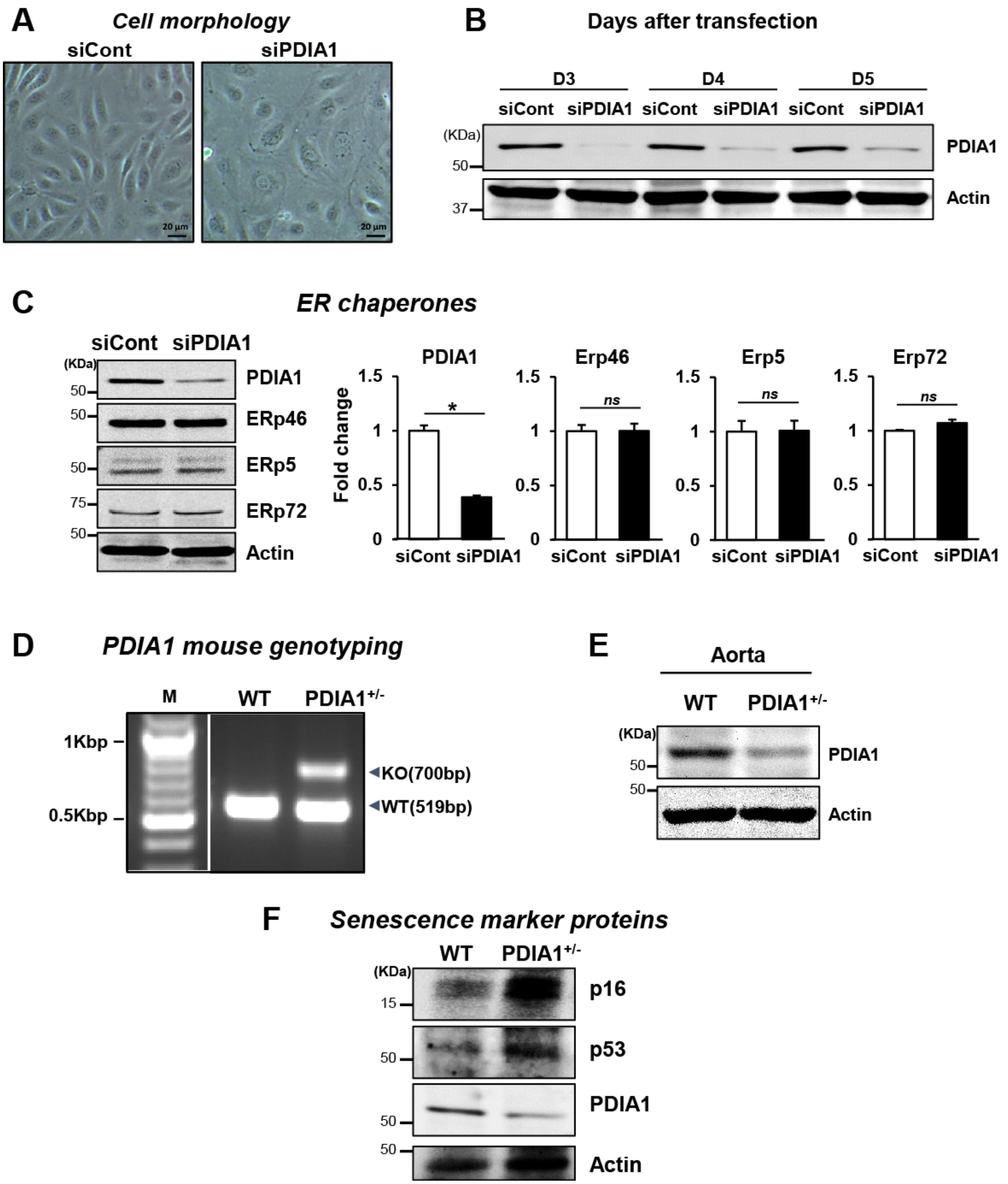

Figure S1. PDIA1 depletion does not alter expression of other ER chaperon proteins in ECs but increases senescence marker proteins in PDIA1$^{+/-}$ mice. A-C, HUVECs were transfected with siControl or siPDIA1, **A,** Cell morphology. **B**, Time course for PDIA1 knockdown efficiency after siPDIA1 transfection. HUVECs transfected with siControl or siPDIA1 were used to measure PDIA1 protein using Western analysis at 3, 4, and 5 days after transfection. **C,** The cell lysates were used to measure ER chaperon proteins (ERp46, ERp5, or ERp72) using Western analysis. **D,** The genotyping result for wild type (WT, control) mice and PDIA1$^{+/-}$ mice. **F**, Western analysis for senescence marker proteins in WT and PDIA1$^{+/-}$ mice. **E,** Reduced PDIA1 protein expression in aorta of PDIA1$^{+/-}$ mice (related with Fig 1H). Data are mean ± SEM or representative of at least three independent experiments. Related to Figure 1.

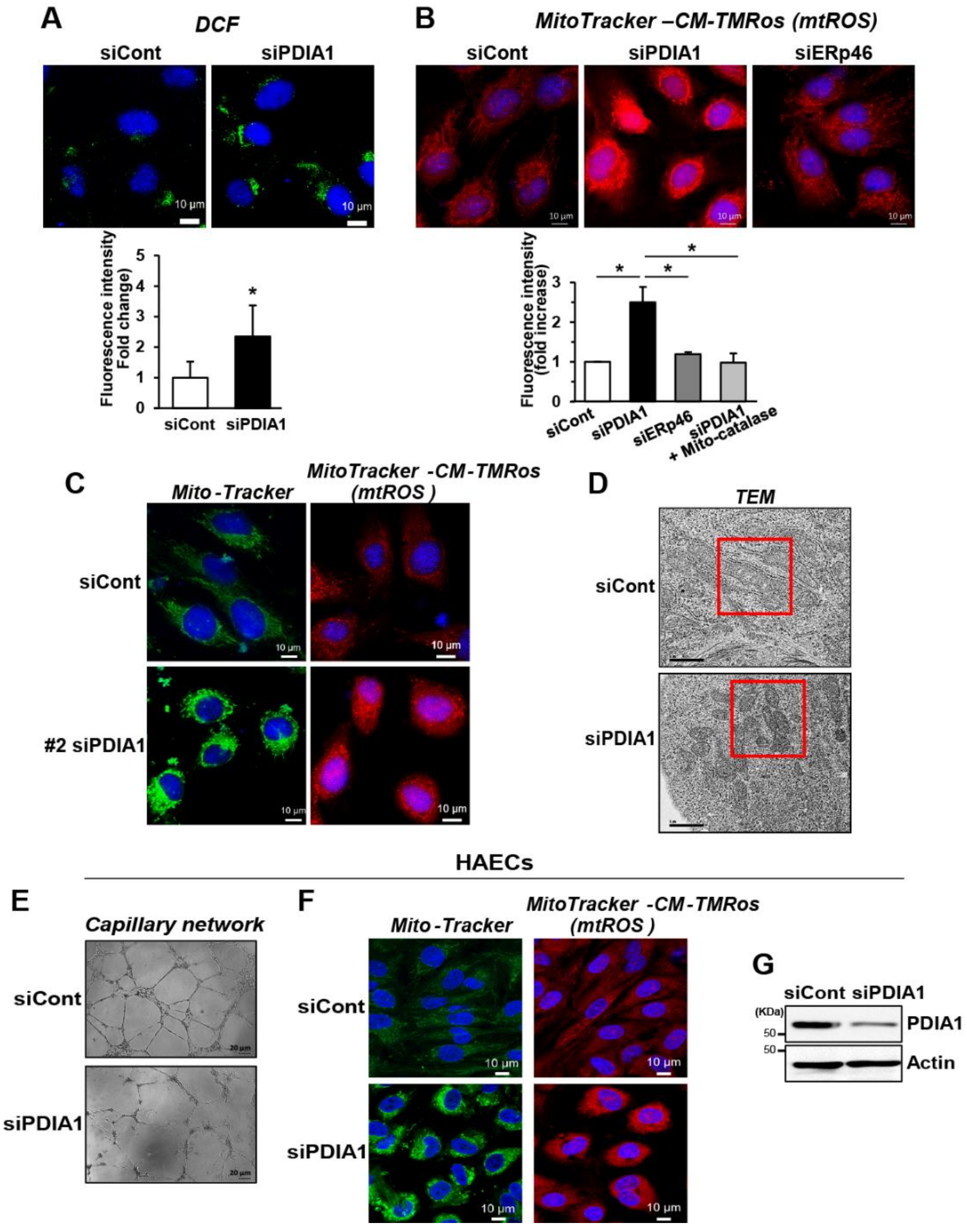

Figure S2. Cytosolic and mitochondrial ROS elevation as well as transmission electron microscopy (TEM) analysis in PDIA1-depleted ECs. A, Cytosolic redox status measured by DCF-DA fluorescence in HUVECs transfected with siControl or siPDIA1. Elevated DCF fluorescence was abolished by PEG-catalase, suggesting that its signal mainly reflects intracellular H_2O_2 (not shown). **B,** Mitochondrial redox status measured by MitoTracker CM-TMRos fluorescence in HUVECs transfected with siControl or siPDIA1 or siERp46; or siPDIA1 with adenovirus expressing mitochondria-targeted catalase (Ad.Mito-catalase). Elevated MitoTracker-CMTMRos fluorescence was abolished by Mito-catalase, suggesting that its signal mainly reflects mtROS. Graph represents the fold increase in fluorescence intensity from the control (siControl group). **C**, Mitochondria fission measured by MitoTracker Green as well as mtROS measured by MitoTracker CM-TMRos in HUVECs transfected with another siPDIA1 with different target sequence (#2). **D,** Low magnification of transmission electron microscopy (TEM) images showing the mitochondrial structure in HUVECs transfected with siControl or siPDIA1. **E-G,** Human aortic ECs (HAECs) are transfected with siControl or siPDIA1. In **E**, Capillary-like network formation on Matrigel. In **F**, Mitochondria morphology visualized by MitoTracker (left) and mtROS levels visualized by MitoTracker CM-TMRos (right). In **G**, PDIA1 or actin (control) protein expression measured by Western analysis, showing the PDIA1 knockdown efficiency. Data are mean ± SEM (A and B) or representative of at least three independent experiments (A-G). Related to Figure 2 and 3.

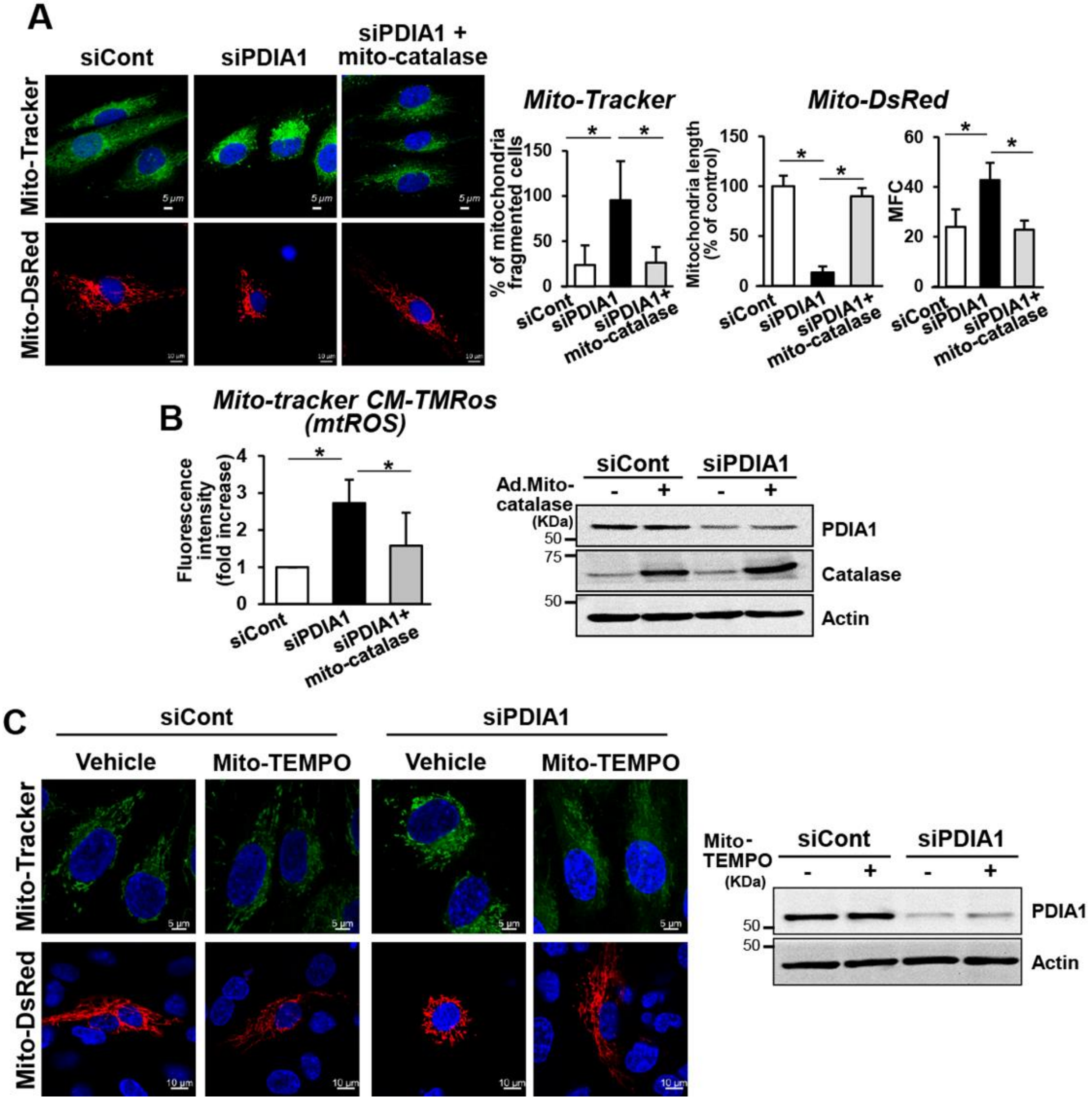

Figure S3. PDIA1 depletion-induced mitochondrial fragmentation and mtROS elevation are inhibited by Mito-catalase and Mito-TEMPO. A and B, HUVECs transfected with siControl or siPDIA1 were infected with Ad.mito-catalase. In **A**, Mitochondrial morphology was visualized by Mito-Tracker (% of mitochondria fragmented cells) or Mito-DsRed (mitochondria length (% of control) or mitochondria fragmentation count (MFC). In **B**, Quantification of mtROS levels measured by MitoTracker CM-TMRos fluorescence. Graph represents the fold increase in fluorescence intensity from the control (siControl group). **C**, HUVECs transfected with siControl or siPDIA1 were treated with 50 μM Mito-TEMPO for 24h and mitochondrial morphology was visualized by Mito-Tracker or Mito-DsRed. The expression of Mito-catalase or PDIA1 was determined by Western analysis. Data are mean ± SEM (n>=3). **p*<0.05 *vs* control siRNA. Related to Figure 2.

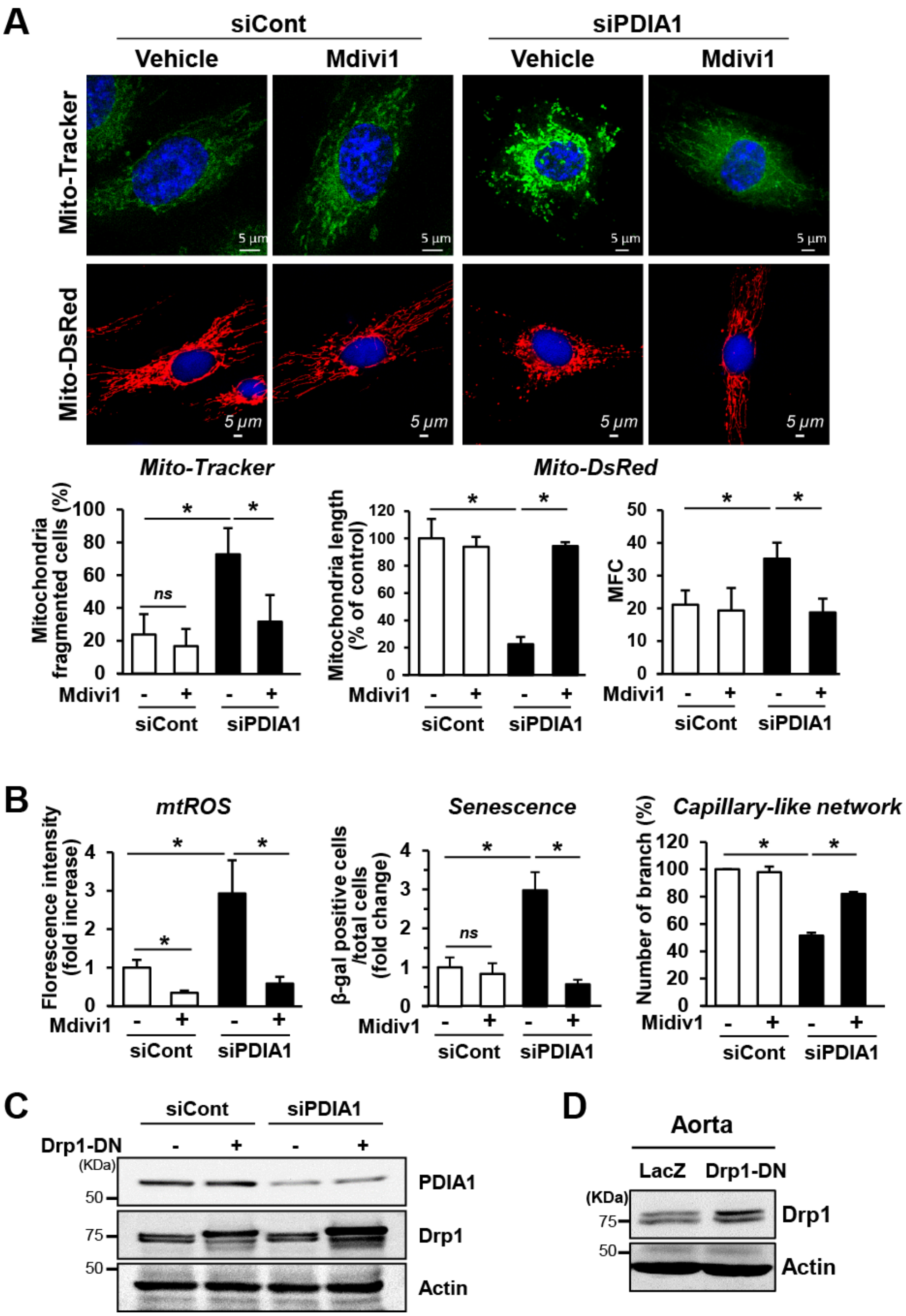

Figure S4. PDIA1 depletion-induced mitochondrial fragmentation, mtROS and endothelial dysfunction are rescued by Drp1 inhibitor, Mdivi1. A, B and C, HUVECs transfected with siControl or siPDIA1 were treated with a specific Drp1 inhibitor, Midivi1 (10 µM) for 24hr (**A** and **B**) or infected with adenovirus expressing Drp1-DN (**C**). In **A**, Mitochondrial morphology was visualized by Mito-Tracker or Mito-DsRed. In **B**, Quantification of mtROS levels measured by MitoTracker CM-TMRos or cell senescence or capillary-like network formation. In **C**, cells were used to measure PDIA1 or Drp1 protein expression using Western analysis. In **D**, Drp1 protein expression in aorta transfected with Ad.LacZ or Ad.Drp1-DN detected by Western blotting (related with Fig 4D). Data are mean ± SEM (n>=3). **p*<0.05 *vs* control siRNA. Related to Figure 4.

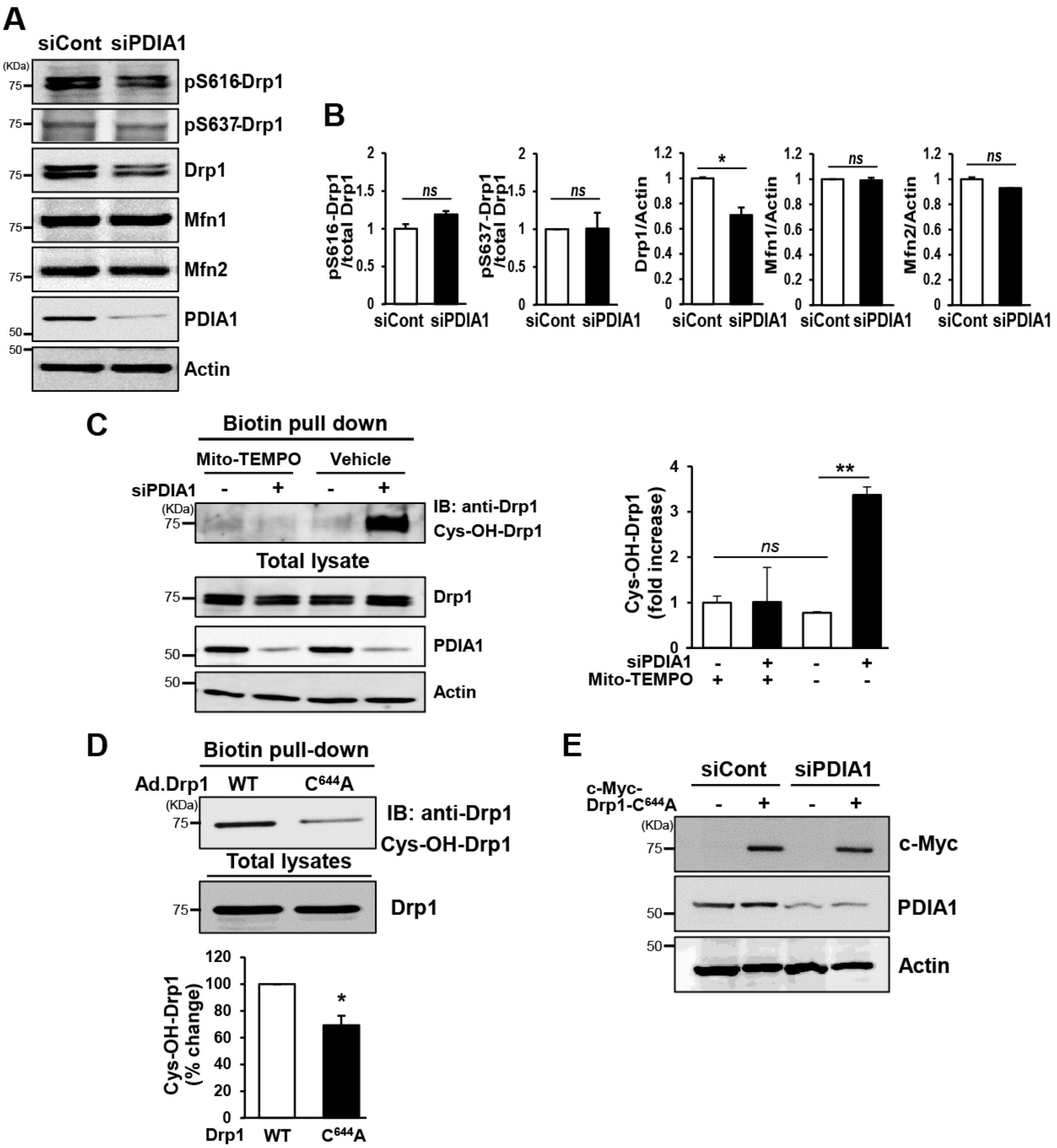

Figure S5. PDIA1 knockdown induces Drp1 sulfenylation without phosphorylation, which was inhibited by Mito-TEMPO or overexpression of Drp1-C^{644}A in ECs. A, B and C, HUVECs were transfected with siControl or siPDIA1. Representative blots (**A**) and quantification (**B**) for p-Ser616-Drp1, protein expression of Drp1, Mfn1, Mfn2, PDIA1 or actin (control) using Western blots analysis. **C**, HUVECs transfected with siControl or siPDIA1 were treat with 50 µM Mito-TEMPO for 24h. DCP-Bio1-labelled lysates were biotin pull-down, followed by immunoblotted (IB) with anti-Drp1 antibody to detect endogenous Drp1 Cys-OH formation. **D**, PDIA1 siRNA-transfected HUVEC was infected with adenovirus expressing c-Myc-Drp1-WT or c-Myc-Drp1-C^{644}A. DCP-Bio1-labeled lysates were biotin pull-down, followed by IB with Drp1 antibody to measure Drp1-Cys-OH formation. **E**, HUVECs transfected with siControl or siPDIA1 were infected with Ad.control or Ad.c-Myc-Drp1-C^{644}A. Lysates were used for Western blot analysis using antibodies indicated. Data are mean ± SEM (n=3-4). *p<0.05, **p<0.001, ***ns***; non-significant. Related to Figure 5.

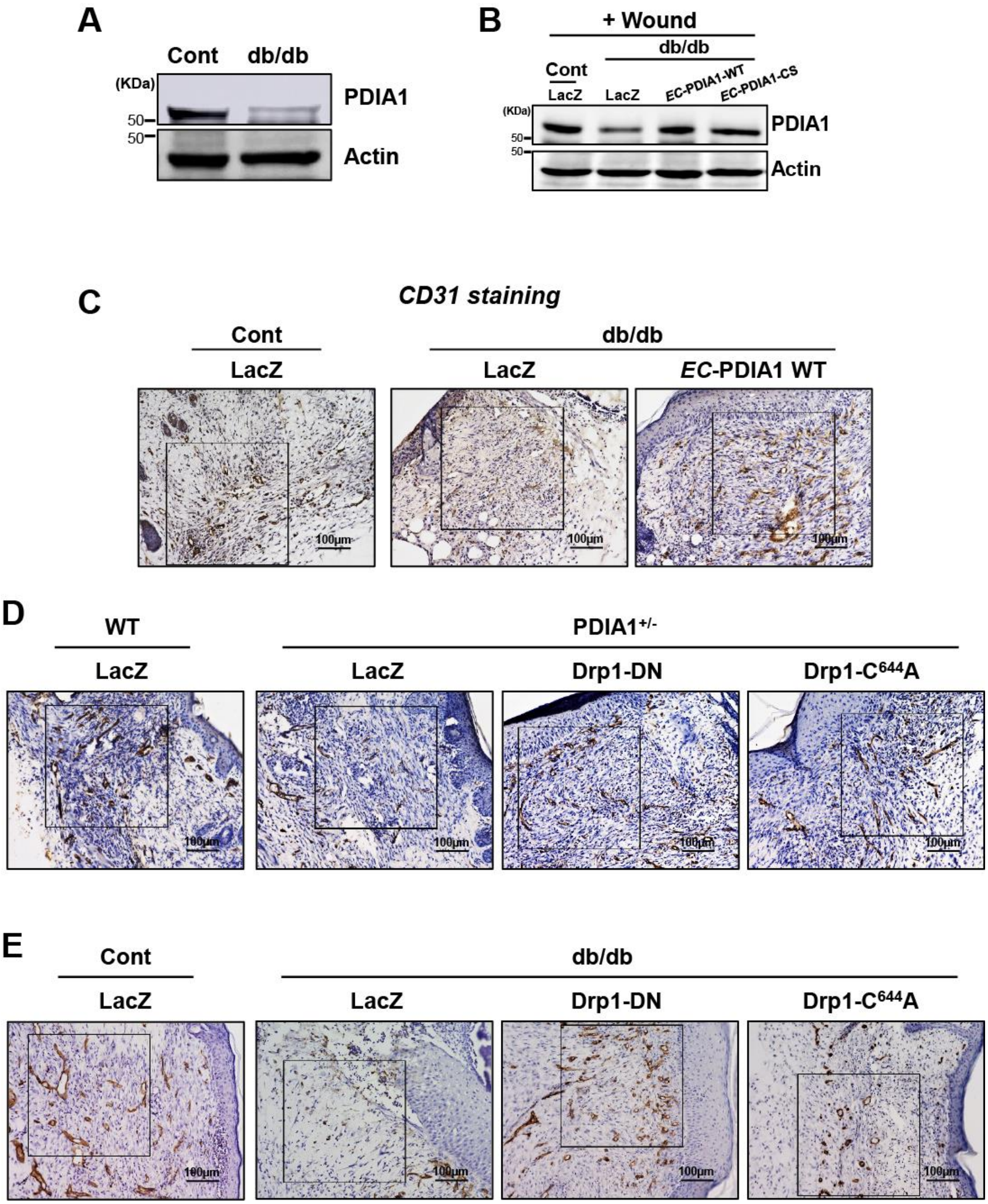

Figure S6. PDIA1 protein is downregulated in db/db mice, and rescue effects of Drp1-DN or Drp1-C^{644}A on impaired angiogenesis in db/db or PDIA1$^{+/-}$mice. A, Western analysis for PDIA1 or actin protein expression in skin of control and db/db mice. **B,** The wounded skin tissues were used to analyze protein expression of PDIA1 or actin (loading control). **C, D, E**, Low magnification of CD31 staining in PDIA1$^{+/-}$ or db/db mice with gene transfer of LacZ (control) or Drp1-DN or Drp1-C^{644}A corresponding to main Figures 7CEG.

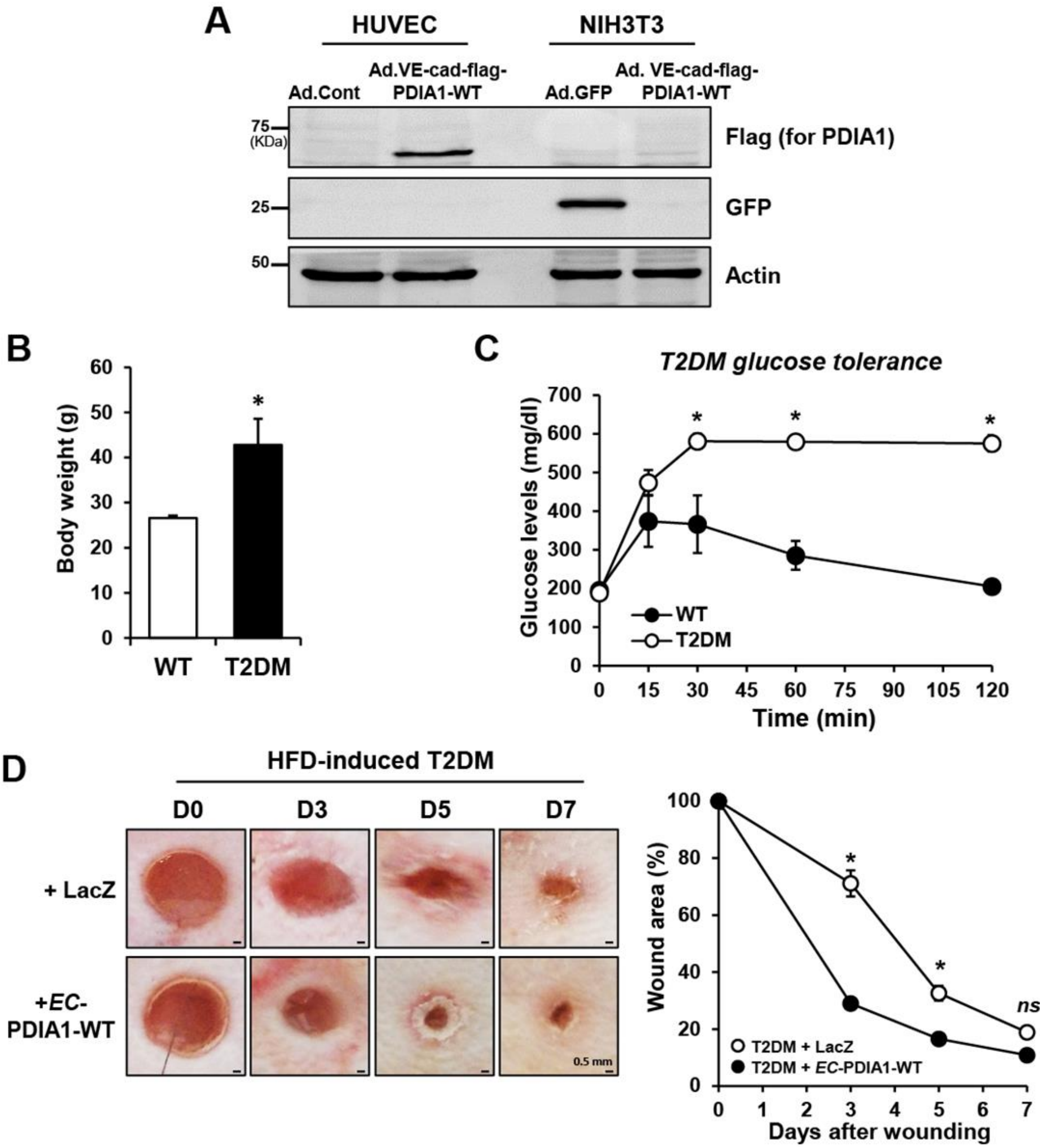

Figure S7. EC-specific PDIA1 gene transfer rescues impaired wound healing in high fat diet (HFD)-induced type2 diabetes mice. A, HUVECs and mouse fibroblast NIH3T3 cells were infected with adenovirus expressing flag-PDIA1 with EC-specific VE-cadherin promoter (Ad.VE-cad-flag-PDIA1-WT) or adenovirus expressing empty vector (Ad.Cont) in HUVECs or GFP (transfection positive control) in NIH3T3 fibroblasts. Western blot analysis using antibodies indicated showing the specific expression of EC-PDIA1 in HUVECs, but not NIH3T3 fibroblasts. **B** and **C**, Characterization of HFD-induced T2DM mice showing increased body weight (**B**) and glucose tolerance (**C**) compared to control (WT) mice. **D**, WT and HFD-induced T2DM mice were wounded on the back skin and wound regions were overlaid with Ad.LacZ, Ad.EC-PDIA1-WT (Ad.VE-cad- flag-PDIA1-WT) and then the wound closing rate was measured for indicated days. Wound area is expressed as % of that measured at right after the wounding (day 0). *P<0.05 vs control group. Related to Figure 7.

Supplemental Material and Methods

Materials

siRNA: #1 siPDIA1 (sense 5'-CAG GGAACCUCUCUGAAGU-3', anti-sense 5'-ACUUCAGAGAGGUUCCCUG-3'), #2 siPDIA1 (sense 5'-UGAGUCUUGAUUUCACCUC-3', anti-sense 5'-GAGGUGAAAUCAAGACUCA-3'), siEndo-PDIA1 (ERp46) (sense 5'-GAAGCUGUGAAGUACCAGGUU-3' anti-sense 5'-AACCUGGUACUUCACAGCUUC-3')

Plasmids: Flag-hPDIA1-WT was from Addgene. Flag-hPDIA1-CS and Flag-rPDIA1-CS [human or rat PDIA1 inactive mutant in which all four active cysteines (53, 56, 397, and 400) were mutated to serine] were constructed using the QuikChange II site-directed mutagenesis kit (Stratagene) according to the manufacturer's instruction. Flag-ratPDIA1-WT (rPDIA1-WT)(Hahm et al., 2013) and pMito-DsRed (Rehman et al., 2012) were previously described. The EC-specific VE-cad-flag-hPDIA1WT (EC-PDIA1-WT) and VE-cad-flag-hPDIA1-CS (EC-PDIA1-CS) were constructed in our laboratory. The N-terminal Venus-PDIA1 was constructed by cloning PDIA1 (Addgene, Plasmid 31382; 18-508aa) with Sal1/BamH1 sites into pFlagVN173N plasmid while the C-terminal venus-Drp1 was constructed by cloning Drp1 with EcoR1/Xho1 sites into pHA-VC155N plasmid (Okur et al., 2014).

Antibodies: Anti- PDIA1(BD Bioscience, 610946), PDIA1(Sigma P7496), PDIA1(Cell Signaling, 3501), ERp46 (Santa Cruz, sc-271667), Drp1(Santa Cruz, sc-271583), DLP1(BD Bioscience, 611112), p21(Santa Cruz, sc-6246), p16(BD Bioscience, 554079), p53(Santa Cruz, sc-6243), Bip1(Cell Signaling, 3177), CHOP(Cell Signaling, 2895), sXBP1(Cell Signaling, 12782), ERp5(Santa Cruz, sc-374494), Erp72(Santa Cruz, sc-292586), c-Myc (Sigma, M4438), Flag (Sigma, F7425), and actin(Santa Cruz, sc-1616) antibodies were used.

Adenovirus: Ad.Drp1-DN (K38A) was previously described (Yu et al., 2006). The EC specific adenovirus including Ad.VE-cad-flag-hPDIA1-WT (Ad.EC-PDIA1-WT) and Ad.VE-cad-flag-hPDIA1-CS (inactive mutant), as well as Ad.c-Myc-Drp1C^{644}A were generated, as previously described with minor modifications (Estrada et al., 2012; Prandini et al., 2005). Ad.mitochondria-targeted catalase (Mito-catalase) (Wang et al., 2011) was obtained from Iowa Gene Transfer Vector Core.

Cell culture: The primary HUVECs (human Umbilical Vein Endothelial Cells) from Lonza (CC-2519, USA) were cultured in EndoGRO (EMD Millipore) supplemented with 5% fetal bovine serum (FBS, Atlanta Biological), and used for experiments until passage 6. The primary HAECs (human Aortic Endothelial Cells) from the Lonza were cultured in EGM-2 (Lonza) supplemented with 2% FBS without VEGF. HEK293T cells from ATCC were cultured in DMEM (Gibco-BRL) with 10% FBS.

Transfection: For siRNA transfection, HUVECs were transfected with 20nM siRNAs using Oigofectamine (Invitrogen, 12252011). For double transfection of siRNA and plasmid DNA, cells were transfected with siRNAs (20nM) and plasmid DNA (3ug for 100 mm dishes) using JetPRIME (Polyplus, USA). For plasmid DNA transfection, cells were transfected with DNA (1~3ug) using polyethylenimine (PEI, Polysciences, USA). After transfection, cells were changed to growth medium and further incubated for 48 h at 37°C before experiments.

Cell growth measurement: After siRNA transfection for 24h, the cells (10,000 cells) were replated on 35mm dish containing 2.5% FBS EndoGro. The growth rate was determined by counting cell numbers using hemocytometer at indicated day.

Senescence-Associated β–Galactosidase (β-gal) staining: β-gal activity at pH6 was measured using Senescence Detection kit (BioVision). Cells were cultured for indicated days by changing media at every other day, and then stained (Shimasaki et al., 2013). The cells were photographed and β-gal positive cells were counted in five different fields/well.

Bromodeoxyuridine (BrdU)-labeled cell proliferation assay: The cells (100,000 cells) were plated on coverslips in 0.5% FBS EndoGro for 8 h to synchronize cells, followed by incubation with 2.5% FBS EndoGro for 6 days and changed media at every other day. For BrdU incorporation, the cells were treated with 5 µM BrdU for 20 h, and then detected using In Situ Cell Proliferation kit, FLUOS (Roche) with minor modification (Wang et al., 2015). The cells were taken photos with fluorescence microscope (Keyence, BZ-X700) using ×40 objective. The percentage of BrdU-labelled cells was determined by counting > 400 nuclei per samples as BrdU-positive/total nuclei.

Capillary like network formation assay: The cells were starved with 0.5% FBS EndoGro medium for overnight. 60,000 cells were seeded on growth factor reduced Matrigel (Corning) coated 48 well plate and incubated for 6 h and then fixed with 4% paraformaldehyde for taking a picture, as we reported (Oshikawa et al., 2012). The capillary tube length, number of branch point, or branch were measured using ImagePro or Image J software, respectively.

Fibrin bead angiogenesis assay: HUVECs infected with lentivirus expressing red fluorescence protein (RFP) were transfected with control or PDIA1 siRNA for 48hr. The capillary-like sprouting fibrin bead assay was performed with minor modifications (Tung and Kitajewski, 2010). Briefly, primary HUVEC and fibroblasts (ATCC) were exposed to M199 medium with 10% FBS. HUVEC were attached to dextran-coated Cytodex 3 beads (GE Healthcare Bio-Sciences, NJ) and embedded at 250 beads/well in 24-well plate in a fibrin clot. Fibroblasts were then seeded as a monolayer over the clot at 1.5×10^5 cells/well. Clots were cultured at standard conditions in EGM-2 medium and media was changed every day for 5-7 days. Bright field and fluorescent images were taken using the Celigo image cytometer (Nexcelom), and sprout number and tip cell number were quantified for 80-100 beads per experimental group. Number of sprouts was the number of independent sprouts extruding from each bead and normalized to the number of beads counted. Tip cell number was number of ends of sprouts included those from branching normalized to number of beads.

Cell cycle analysis: Cells in each cell cycle were analyzed using FACS analysis (Becton Dickinson, USA) and Modfit software, as described (Shimasaki et al., 2013). Briefly, siRNA transfected-cells were incubated for 48 h and the cells were trypsinized (retaining all floating cells) and fixed with 70% ethanol at 4 °C for overnight. Cells were then incubated at room temperature for 30 min with 50 mg/mL propidium iodide (PI; Sigma) and 5 mg/mL RNase A (QIAGEN). The number of cells in each cell cycle were evaluated using FACS Calibur system.

Mitochondria respiratory capacity (O_2 consumption rate, OCR) and Extracellular acidification rate (ECAR): Transfected cells (90~95% confluence, 200-250K cells/well) were plated in XF24 extracellular flux assay kits (24 well plate, 100850-001) at one day before measurement using Seahorse analyzer which measure the response to the respiratory chain inhibitors such as Oligomycin, FCCP, rotenone/antimycin. For O_2 consumption rate (OCR) measurement, cell culture media was changed to modified XF assay medium (100965-000, unbuffered, glucose free, and pyruvate free) containing low glucose (0.1% (W/V), ~5.5 mM). For extracellular acidification rate (ECAR) for glycolysis flux measurement, cell culture media was changed to modified XF assay medium containing 2 mM glutamine. After 1h incubation at 37 ºC without CO_2, the OCR and ECAR were measured using Seahorse according to manufacturer's instruction (XF Cell Mito stress test kit;103015-100 and glycolysis stress test kit;103020-100). The data were expressed by the percent control (100%) after normalizing with the cell number.

Mitochondria structure analysis: We used two different methods to visualize and analyze mitochondrial structure in live cells. First, we used Mito-DsRed mammalian expression vector with the mitochondrial targeting sequence fused to the 5' end of the DsRed vector with CMV promoter (Karbowski et al., 2002; Rehman et al., 2012). Mito-DsRed labels mitochondria in living cells with little photobreaching and enables us to measure mitochondria length precisely. Second, we used MitoTracker Green FM (Invitrogen, M-7514) fluorescence dye to stain active mitochondria which shows the mitochondria morphology (Torres-Gonzalez et al., 2014). Cells were incubated with 500 nM MitoTracker Green FM for 30 min or transfected with Mito-DsRed plasmid, and then images were taken by super resolution microscopy (Nikon, magnification x100) or confocal microscopy (Zeiss LSM710, magnification x63). Mitochondrial super resolution images were obtained using a Nikon Instruments N-SIM Super Resolution Microscope System equipped with a CFI60 Apochromat 100X oil immersion Objective Lens (N.A.1.49). Images were captured with an Ixon DU897 Ultra EMCCD camera (200 ms exposure time). GFP (for MitoTracker Green) was excited with the 488 nm laser and RFP (for Mito-DsRed) was excited with the 561 nm laser. NIS-Elements software was used for image acquisition and reconstruction. Mito-DsRed plasmid-transfected ECs showed much clearer mitochondria structure to measure mitochondria length than MitoTracker Green. Therefore, we measured the mitochondrial length in Mito-DsRed-expressing individual cell from mitochondria initial point (near nuclear) to endpoint using Image Pro from at least 50 mitochondria in a triplicate and blinded manner (Brooks et al., 2007). Data are expressed by the relative values compared to control group (100%). Acquired Mito-DsRed images were background subtracted, filtered (median), thresholded, and binarized to identify mitochondrial fission using ImageJ (Koopman et al., 2005; Mortiboys et al., 2008; Rehman et al., 2012). Following calculation for mitochondrial fission count (MFC, number of particles/total mitochondria pixels X1000) involves the ratio between the number of mitochondrial fragments and total mitochondrial area. For MitoTracker Green-stained cells, mitochondria

fragmented cells were counted using Image J and expressed by the % ratio of mitochondria fragmented cells/total cells in the same filed (around 5~8 cells/field, x63 magnification) at least in the five fields in a triplicate. We classified the fragmented mitochondria as shortened, punctate, and sometimes rounded shape, while intact (filamentous) mitochondria as a long tubular structure. The morphometric analyses were performed to calculate the degree of mitochondrial fission.

Live cell imaging for mitochondria dynamics: Cells were incubated with 50 nM TMRM (Invitrogen, T-668) for 30 min, and cells in Hank's Balanced Salt solution (HBSS)(Gibco #14025-092) were used to monitor mitochondria dynamics by recording TMRM fluorescence in live cells using confocal microscopy (Zeiss LSM710, x100) as reported (Papanicolaou et al., 2011). At low concentrations (10-50 nM range) of TMRM, it has no effects on mitochondrial respirations (Scaduto and Grotyohann, 1999) and has low toxicity and photobreaching (Ward et al., 2010). Thus, during the short period of recording for 5min, we confirmed that cells showed either no toxic effects or inducing mitochondria permeability transition pore opining. The setting of the microscopy was followed; Time series 150 frames, scaling (per pixel) 0.09 µm X 0.09 µm, Image size (scale) 45.00 µm X 45.00 µm, Bit depth 8 bit, objective alpha plan-fluor 100x/1.45, excitation wavelength 543nm, emission wavelength 580nm.

Mitochondrial fusion analysis using mitochondria-targeted photoactivatable-GFP (mito-PA-GFP): HUVECs were transfected with siRNA for control and PDIA1 for 24 h and then infected with adenovirus expressing mito-PA-GFP (Ad.mito-PA-GFP) for 24h. The cells were serum starved with 0.5% FBS EndoGro for 3h and incubated with 700 nM Mito-tracker Red for 15 min to visualize the mitochondria structure. The live cell imaging was conducted under 37°C, 5% CO_2 with humidity. The minimum region (400µm^2) defined by the apparatus (LSM780, Zeiss, magnification x63 with x2 zoom, Alpha plan-Apo (oil) NA1.46) was photoactivated using 405nm laser at 100% laser power briefly (Karbowski et al., 2014; Lovy et al., 2012). GFP signal was imaged using 488nm laser and the Mito-tracker red was determined using 543nm laser. After photoactivation, images were obtained at 10 sec intervals for 1000-1200 sec (100-120 frames). A decrease of mean intensity of GFP fluorescence was measured as fusion (Arasaki et al., 2015; Hong et al., 2013; Motori et al., 2013; Rehman et al., 2012).

Mitochondria redox status (ROS) measurement: To measure mitochondria redox status, ECs were incubated with 5 µM MitoSox Red (Invitrogen) for 10 min, or 800 nM MitoTracker Orange CMTMRos (Invitrogen, M7511) for 30 min, and fluorescence intensity in cells were measured using confocal microscopy (Zeiss LSM810, x63) as we reported (Kim et al., 2017). We confirmed that MitoSox and MitoTracker CMTMRos fluorescence signals were abolished by mitochondria-targeted SOD mimetic, Mito-TEMPO (Enzo) and overexpression of mitochondria-targeted catalase (mito-catalase), respectively, suggesting that they measure mitochondria-derived ROS (mtROS) levels. We measured the fluorescence intensity from each sample among different groups under the same setting using Image J. Relative fluorescence intensity was expressed as fold increase from the control (basal siControl-transfected cells).

Transmission electron microscopy (TEM): Cells were washed with Sorenson buffer (pH 7.4) and fixed with 2% glutaraldehyde in Sorenson buffer (pH 7.4) for 30 min on 4ºC. The images were taken by JEM-1220 (JEOL) at electron microscopy core facility at University of Illinois at Chicago.

DCP-Bio1 assay to detect Cys-OH formed (sulfenylated) proteins: To measure sulfenic acid (Cys-OH) formation (sulfenylation) of proteins, cells were lysed in degassed-specific lysis buffer [50 mM HEPES, pH7.0 at room temperature, 5 mM EDTA, 50 mM NaCl, 50 mM NaF, 1 mM Na_3VO_4, 10 mM sodium pyrophosphate, 5 mM Iodoacetamide (IAA), 100 µM DTPA, 1% Triton-X-100, protease inhibitor, 200 unit/mL catalase (Calbiochem), 200 µM DCP-Bio1 (KaraFast, USA)] and then DCP-Bio1-bound proteins were pulled down with streptavidin beads (Thermo scientific, USA) for overnight at 4 ºC. DCP-Bio1-bound Cys-OH formed, sulfenylated-proteins were determined by immunoblotting with specific antibodies, as reported (Kaplan et al., 2011; Oshikawa et al., 2010; Poole and Nelson, 2008).

PDI reductase activity assay: Cells or tissues were extracted in KPi buffer (50 mM KPi, pH7.4, 300 mM KBr, protease inhibitor cocktail) and 10ug protein was used to measure PDI reductase activity using ProteoStat PDI assay kit by following manufacturer's instruction (ENZ-51024)(Tufo et al., 2014).

Drp1 GTPase activity assay: Cells were lysed in lysis buffer (50nM HEPES, pH7.5, 120 mM NaCl, 5mM EDTA, 10mM Na pyrophosphate, 50mM NaF, 1mM Na_3VO_4, 1% Triton X-100, protease inhibitors) and total cell lysate

(1mg) was used for immunoprecipitation using Drp1 antibody (BD Bioscience) for overnight at 4ºC. Anti-Drp1 immunoprecipitates were incubated with 50 uL protein A/G beads (Santa Cruz, sc-2003) for 2h, and beads were washed three times with lysis buffer, and further washed three times with GTPase buffer (50 mM Tris, pH7.5, 2.5 mM $MgCl_2$, 0.02% β-Mercaptoethanol) as described previously (Qi et al., 2013). The GTPase activity was measured using the GTPase assay kit by following manufacturer's instruction (NUVUS, Innova Bioscience).

BiFc (Bimolecular Fluorescence Complementation) assay: COS1 cells were transfected with DNAs (each 250~500 ng) using PEI as follows; ***negative control***: N-terminal Venus-PDIA1+C-terminal Venus-negative peptide+pECFP, ***PDIA1+Drp1***: N-terminal venus-PDIA1+C-terminal venus-Drp1+pECFP for 24 h. The positive YFP signals showing interaction between PDIA1 and Drp1 were taken by confocal microscope, as reported (Okur et al., 2014). pECFP was used for transfection positive control.

Immunofluorescence analysis using confocal microscopy: Cells (over 60% confluence) growing on coverslips in 6 well plates were fixed with 4% paraformaldehyde for 10 min and then blocked with blocking buffer (1X PBS, 5% normal goat serum, and 0.3% Triton X-100) for 1 h. Cells were incubated with the primary antibodies (PDIA1; Sigma P7496, Drp1; BD Bioscience,611112) in dilution solution (1X PBS, 1% BSA, and 0.3% Triton X-100) for overnight at 4 ºC and then incubated with secondary antibodies (Alexa Fluor Plus 488(A32731) or 546(A11030), Invitrogen) for 1h at room temperature. The images were taken using confocal microscopy (x63).

Western blot analysis and Immunoprecipitation assay: Cells were lysed in buffer [50 mM HEPES (pH 7.4), 5 mM EDTA, 100 mM NaCl, 1% Triton X-100, protease inhibitors (10 μg/ml aprotinin, 1 mmol/L phenylmethylsulfonyl fluoride, 10 μg/ml leupeptin) and phosphatase inhibitors (50 mmol/L sodium fluoride, 1 mmol/L sodium orthovanadate, 10 mmol/L sodium pyrophosphate)]. Lysates with or without immunoprecipitation were used for Western blotting, as we reported (Oshikawa et al., 2010).

Immunohistochemistry for CD31 staining: Mice were sacrificed at 7–11 days after wound punch, and skin wounded tissue were harvested, fixed with 4% PFA for overnight at 4 ºC, and followed by sucrose dehydration and OCT embedding. Capillary density in wound skin tissues was determined as we reported (Das et al., 2016). Images were taken using fluorescence microscope (Keyence, BZ-X700) with a ×20 objective.

Endothelium-dependent or independent vasorelaxation: Aortas of WT or $PDIA1^{+/-}$ mice were used for measuring acetylcholine (Ach)- or sodiumnitroprusside (SNP)-induced vasorelaxation, as we reported (Sudhahar et al., 2013**).** To evaluate rescue effect of Drp1-DN on impaired endothelium-dependent relaxation of $PDIA1^{+/-}$ mice, aortas were infected with Ad.LacZ or Ad.Drp1-DN for 1h at 37ºC and further incubated for 20 h at 4ºC before experiments.

Supplemental References

Arasaki, K., Shimizu, H., Mogari, H., Nishida, N., Hirota, N., Furuno, A., Kudo, Y., Baba, M., Baba, N., Cheng, J., *et al.* (2015). A role for the ancient SNARE syntaxin 17 in regulating mitochondrial division. Dev Cell *32*, 304-317.
Brooks, C., Wei, Q., Feng, L., Dong, G., Tao, Y., Mei, L., Xie, Z.J., and Dong, Z. (2007). Bak regulates mitochondrial morphology and pathology during apoptosis by interacting with mitofusins. Proceedings of the National Academy of Sciences of the United States of America *104*, 11649-11654.
Das, A., Sudhahar, V., Chen, G.F., Kim, H.W., Youn, S.W., Finney, L., Vogt, S., Yang, J., Kweon, J., Surenkhuu, B., *et al.* (2016). Endothelial Antioxidant-1: a Key Mediator of Copper-dependent Wound Healing in vivo. Sci Rep *6*, 33783.
Estrada, I.A., Donthamsetty, R., Debski, P., Zhou, M.H., Zhang, S.L., Yuan, J.X., Han, W., and Makino, A. (2012). STIM1 restores coronary endothelial function in type 1 diabetic mice. Circ Res *111*, 1166-1175.
Hahm, E., Li, J., Kim, K., Huh, S., Rogelj, S., and Cho, J. (2013). Extracellular protein disulfide isomerase regulates ligand-binding activity of alphaMbeta2 integrin and neutrophil recruitment during vascular inflammation. Blood *121*, 3789-3800, S3781-3715.
Hong, Z., Kutty, S., Toth, P.T., Marsboom, G., Hammel, J.M., Chamberlain, C., Ryan, J.J., Zhang, H.J., Sharp, W.W., Morrow, E., *et al.* (2013). Role of dynamin-related protein 1 (Drp1)-mediated mitochondrial fission in oxygen sensing and constriction of the ductus arteriosus. Circ Res *112*, 802-815.
Kaplan, N., Urao, N., Furuta, E., Kim, S.J., Razvi, M., Nakamura, Y., McKinney, R.D., Poole, L.B., Fukai, T., and Ushio-Fukai, M. (2011). Localized cysteine sulfenic acid formation by vascular endothelial growth factor: role in endothelial cell migration and angiogenesis. Free Radic Res *45*, 1124-1135.
Karbowski, M., Cleland, M.M., and Roelofs, B.A. (2014). Photoactivatable green fluorescent protein-based visualization and quantification of mitochondrial fusion and mitochondrial network complexity in living cells. Methods Enzymol *547*, 57-73.
Karbowski, M., Lee, Y.J., Gaume, B., Jeong, S.Y., Frank, S., Nechushtan, A., Santel, A., Fuller, M., Smith, C.L., and Youle, R.J. (2002). Spatial and temporal association of Bax with mitochondrial fission sites, Drp1, and Mfn2 during apoptosis. J Cell Biol *159*, 931-938.
Kim, Y.M., Kim, S.J., Tatsunami, R., Yamamura, H., Fukai, T., and Ushio-Fukai, M. (2017). ROS-induced ROS release orchestrated by Nox4, Nox2, and mitochondria in VEGF signaling and angiogenesis. Am J Physiol Cell Physiol *312*, C749-C764.
Koopman, W.J., Visch, H.J., Verkaart, S., van den Heuvel, L.W., Smeitink, J.A., and Willems, P.H. (2005). Mitochondrial network complexity and pathological decrease in complex I activity are tightly correlated in isolated human complex I deficiency. Am J Physiol Cell Physiol *289*, C881-890.
Lovy, A., Molina, A.J., Cerqueira, F.M., Trudeau, K., and Shirihai, O.S. (2012). A faster, high resolution, mtPA-GFP-based mitochondrial fusion assay acquiring kinetic data of multiple cells in parallel using confocal microscopy. J Vis Exp, e3991.
Mortiboys, H., Thomas, K.J., Koopman, W.J., Klaffke, S., Abou-Sleiman, P., Olpin, S., Wood, N.W., Willems, P.H., Smeitink, J.A., Cookson, M.R., *et al.* (2008). Mitochondrial function and morphology are impaired in parkin-mutant fibroblasts. Ann Neurol *64*, 555-565.
Motori, E., Puyal, J., Toni, N., Ghanem, A., Angeloni, C., Malaguti, M., Cantelli-Forti, G., Berninger, B., Conzelmann, K.K., Gotz, M., *et al.* (2013). Inflammation-induced alteration of astrocyte mitochondrial dynamics requires autophagy for mitochondrial network maintenance. Cell Metab *18*, 844-859.
Okur, M.N., Russo, A., and O'Bryan, J.P. (2014). Receptor tyrosine kinase ubiquitylation involves the dynamic regulation of Cbl-Spry2 by intersectin 1 and the Shp2 tyrosine phosphatase. Mol Cell Biol *34*, 271-279.
Oshikawa, J., Kim, S.J., Furuta, E., Caliceti, C., Chen, G.F., McKinney, R.D., Kuhr, F., Levitan, I., Fukai, T., and Ushio-Fukai, M. (2012). Novel role of p66Shc in ROS-dependent VEGF signaling and angiogenesis in endothelial cells. Am J Physiol Heart Circ Physiol *302*, H724-732.
Oshikawa, J., Urao, N., Kim, H.W., Kaplan, N., Razvi, M., McKinney, R., Poole, L.B., Fukai, T., and Ushio-Fukai, M. (2010). Extracellular SOD-derived H2O2 promotes VEGF signaling in caveolae/lipid rafts and post-ischemic angiogenesis in mice. PLoS One *5*, e10189.
Papanicolaou, K.N., Khairallah, R.J., Ngoh, G.A., Chikando, A., Luptak, I., O'Shea, K.M., Riley, D.D., Lugus, J.J., Colucci, W.S., Lederer, W.J., *et al.* (2011). Mitofusin-2 maintains mitochondrial structure and contributes to stress-induced permeability transition in cardiac myocytes. Mol Cell Biol *31*, 1309-1328.
Poole, L.B., and Nelson, K.J. (2008). Discovering mechanisms of signaling-mediated cysteine oxidation. Curr Opin Chem Biol *12*, 18-24.

Prandini, M.H., Dreher, I., Bouillot, S., Benkerri, S., Moll, T., and Huber, P. (2005). The human VE-cadherin promoter is subjected to organ-specific regulation and is activated in tumour angiogenesis. Oncogene *24*, 2992-3001.
Qi, X., Qvit, N., Su, Y.C., and Mochly-Rosen, D. (2013). A novel Drp1 inhibitor diminishes aberrant mitochondrial fission and neurotoxicity. J Cell Sci *126*, 789-802.
Rehman, J., Zhang, H.J., Toth, P.T., Zhang, Y., Marsboom, G., Hong, Z., Salgia, R., Husain, A.N., Wietholt, C., and Archer, S.L. (2012). Inhibition of mitochondrial fission prevents cell cycle progression in lung cancer. FASEB J *26*, 2175-2186.
Scaduto, R.C., Jr., and Grotyohann, L.W. (1999). Measurement of mitochondrial membrane potential using fluorescent rhodamine derivatives. Biophys J *76*, 469-477.
Shimasaki, Y., Pan, N., Messina, L.M., Li, C., Chen, K., Liu, L., Cooper, M.P., Vita, J.A., and Keaney, J.F., Jr. (2013). Uncoupling protein 2 impacts endothelial phenotype via p53-mediated control of mitochondrial dynamics. Circ Res *113*, 891-901.
Sudhahar, V., Urao, N., Oshikawa, J., McKinney, R.D., Llanos, R.M., Mercer, J.F., Ushio-Fukai, M., and Fukai, T. (2013). Copper transporter ATP7A protects against endothelial dysfunction in type 1 diabetic mice by regulating extracellular superoxide dismutase. Diabetes *62*, 3839-3850.
Torres-Gonzalez, M., Gawlowski, T., Kocalis, H., Scott, B.T., and Dillmann, W.H. (2014). Mitochondrial 8-oxoguanine glycosylase decreases mitochondrial fragmentation and improves mitochondrial function in H9C2 cells under oxidative stress conditions. Am J Physiol Cell Physiol *306*, C221-229.
Tufo, G., Jones, A.W., Wang, Z., Hamelin, J., Tajeddine, N., Esposti, D.D., Martel, C., Boursier, C., Gallerne, C., Migdal, C.*, et al.* (2014). The protein disulfide isomerases PDIA4 and PDIA6 mediate resistance to cisplatin-induced cell death in lung adenocarcinoma. Cell Death Differ *21*, 685-695.
Tung, J.J., and Kitajewski, J. (2010). Chloride intracellular channel 1 functions in endothelial cell growth and migration. Journal of angiogenesis research *2*, 23.
Wang, S., Amato, K.R., Song, W., Youngblood, V., Lee, K., Boothby, M., Brantley-Sieders, D.M., and Chen, J. (2015). Regulation of endothelial cell proliferation and vascular assembly through distinct mTORC2 signaling pathways. Mol Cell Biol *35*, 1299-1313.
Wang, Y., Zang, Q.S., Liu, Z., Wu, Q., Maass, D., Dulan, G., Shaul, P.W., Melito, L., Frantz, D.E., Kilgore, J.A.*, et al.* (2011). Regulation of VEGF-induced endothelial cell migration by mitochondrial reactive oxygen species. Am J Physiol Cell Physiol *301*, C695-704.
Ward, M.W., Concannon, C.G., Whyte, J., Walsh, C.M., Corley, B., and Prehn, J.H. (2010). The amyloid precursor protein intracellular domain(AICD) disrupts actin dynamics and mitochondrial bioenergetics. J Neurochem *113*, 275-284.
Yu, T., Robotham, J.L., and Yoon, Y. (2006). Increased production of reactive oxygen species in hyperglycemic conditions requires dynamic change of mitochondrial morphology. Proceedings of the National Academy of Sciences of the United States of America *103*, 2653-2658.
